# Health behaviour changes around transitions into and out of unpaid caregiving in the UK: A longitudinal study

**DOI:** 10.1016/j.ssmph.2026.101975

**Published:** 2026-09-21

**Authors:** Enrico Pfeifer, Rebecca Lacey, Baowen Xue, Hynek Pikhart, Anne McMunn

**Affiliations:** a Institute of Epidemiology and Healthcare, University College London, 1-19 Torrington Place, London, WC1E 7HB, United Kingdom; b Population Health Research Institute, City St George's, University of London, Cranmer Terrace, London, SW17 0RE, United Kingdom

**Keywords:** Unpaid caregiving, Health behaviours, Physical activity, Smoking, Alcohol use, Longitudinal study, UKHLS, Fixed effects, Propensity score matching

## Abstract

**Background:**

While unpaid caregiving has become an increasing public health concern, less is known about its association with modifiable health behaviours across the lifecourse. This study examined how transitions into and out of unpaid caregiving influence physical activity, diet, smoking, and alcohol consumption among UK adults aged 16 years and older.

**Methods:**

We used longitudinal data from up to 35,779 participants from the UK Household Longitudinal Study (UKHLS) spanning 2013 to 2023. Fixed effects models were used to estimate changes in health behaviour when people transition in or out of caregiving. As a robustness check, we applied piecewise growth curve models on a propensity score matched sample to model the trajectory of behaviours before, during and after the transition.

**Results:**

Transitioning into caregiving was associated with decreased physical inactivity while caregiving exit was linked to a rise in physical inactivity, although this may reflect caregiving-related physical tasks rather than health-enhancing leisure activity. Entry into caregiving was also associated with an increased probability of smoking particularly among adults aged 16–49, while changes in diet were not statistically significant. Findings for problematic alcohol use were inconsistent between the fixed effect models and piecewise regression.

**Conclusions:**

Caregiving transitions are associated with both beneficial and adverse changes in health behaviours. Caution is warranted in interpreting potential positive health behaviour changes which may reflect a burden of caregiving rather than healthy lifestyle choices. The findings underscore the need for targeted public health interventions that support unpaid caregivers.

## Highlights

• First UK longitudinal study on health behaviours during caregiving transitions.
• Uses lifecourse framework to examine adults aged 16+ over time.
• Investigates both entry and exit within a unified analytical approach.
• Caregiving entry linked to more physical activity but higher smoking rates.
• Caregiving exit associated with less physical activity.

## 1 Introduction

Population ageing and the growing prevalence of chronic conditions have contributed to increased demand for care, much of which is provided unpaid by family, friends, or neighbours. In the UK, around 6.5 million adults provided unpaid care in 2020 (Office For National Statistics (ONS), 2023; Petrillo, Zhang, & Bennett, 2024), with a substantial proportion entering and exiting caregiving each year (Petrillo & Bennett, 2022). In the UK, formal long-term care provision is means-tested and often limited in scope, meaning that a substantial share of care needs is met informally by family and friends (Gulland, 2024; van Damme et al., 2025). Early research linked caregiving with stress, poor health, and increased mortality (Pinquart & Sörensen, 2003; Vitaliano et al., 2003). Although some later studies suggest reduced mortality among caregivers compared to non-caregivers (Mehri et al., 2021; Roth et al., 2015), prospective longitudinal evidence consistently points to declines in psychological well-being following the transition into caregiving, particularly among women and those providing more intensive care (Krämer & Bleidorn, 2024; Lacey et al., 2024). Caregiving outcomes vary by intensity, setting, and the caregiver-care recipient relationship. High-intensity caregivers generally experience more negative health outcomes including higher psychological stress and poorer mental health while lower intensity caregiving is associated with fewer adverse effects, although evidence for positive well-being effects remains limited in prospective studies (Brown & Brown, 2014; Krämer & Bleidorn, 2024). Women more frequently report negative impacts, reflecting greater caregiving demands and gendered expectation in unpaid care provision (Lacey et al., 2019; Pinquart & Sörensen, 2006). Most research has focused on older caregivers and clinical outcomes, neglecting the behavioural mechanisms that link caregiving to health, particularly in younger adults. Yet early caregiving may shape health trajectories across the lifecourse (Koumoutzis et al., 2020; Lacey et al., 2024). Health behaviours such as physical activity, diet, smoking, and alcohol use may serve as modifiable pathways connecting caregiving to long-term health outcomes (Ehsan et al., 2019; Halfon & Hochstein, 2002). For instance, caregivers who take up smoking in response to stress might become less physically active, or adopt less healthy dietary patterns may face elevated cardiovascular and cancer risk long after the caregiving role has ended.

Two theoretical perspectives are central to this study. Firstly, lifecourse theory emphasises that the timing and context of transitions shape their consequences (Bartley, 2017; Dannefer, 2003). Caregiving transitions may disrupt established routines and health behaviours differently depending on the life stage at which they occur and the resources available to the individual. Secondly, role theory complements this by conceptualising caregiving as a process of identity change that can generate psychological strain (Bruhn & Rebach, 2014; Montgomery et al., 2007), particularly when the role becomes prolonged (Aneshensel et al., 1993; Fauth et al., 2015). Together, these perspectives suggest that caregiving may affect health behaviours through the channels proposed by the COM-B behaviour change model (Michie et al., 2011). Stress and psychological strain may alter motivation, prompting maladaptive coping such as smoking or increased alcohol use while time demands and financial pressures may limit opportunities for physical activity or healthy eating. Conversely, caregiving may also enhance capability, as managing the care recipient's condition may increase caregiver's health knowledge and awareness. Such 'teachable moments' may motivate caregivers to adopt healthier behaviours themselves (Cohen et al., 2011).

Despite growing interest in understanding the mechanisms through which unpaid caregiving may influence health, evidence on its association with modifiable health behaviours remains mixed. This inconsistency likely reflects differences in study design, variation in how caregiving and health behaviours are measured, small non-representative samples, and a predominant focus on older or clinical caregiving populations. Most studies are cross-sectional and based on older populations (Armstrong-Carter et al., 2022; Castro et al., 2007; Diaz, 2020; Fredman et al., 2006; Fuchs et al., 2023; Garcia-Mayor et al., 2021; Gonzalez-de Paz et al., 2016; Gottschalk et al., 2020; Hoffman et al., 2012; Horner-Johnson et al., 2015; Jacob et al., 2020; Keller & Ohlendorf, 2024; Kilmer et al., 2024; Litzelman et al., 2018; Reeves et al., 2012; Stacey et al., 2019; Yamashita et al., 2018). Across studies, findings on physical activity are inconsistent, with some studies reporting higher activity among caregivers (Gottschalk et al., 2020; Jacob et al., 2020; Reeves et al., 2012; Zan & Shin, 2022) and others suggesting reduced or unchanged levels (Farrugia et al., 2019; Fredman et al., 2006; Hiyoshi et al., 2023; Taniguchi et al., 2025; Yamashita et al., 2018) but physical activity measures varied widely across studies. Evidence on diet and alcohol use is similarly inconclusive, with several studies indicating less favourable dietary patterns (Garcia-Mayor et al., 2021; Hiyoshi et al., 2023; Hossain et al., 2021) but longitudinal evidence suggesting that caregiving was associated with an increase in problematic drinking (Hiyoshi et al., 2023; Taniguchi et al., 2025). Smoking also appears more common among caregivers (Gottschalk et al., 2020; Grenard et al., 2020; Kilmer et al., 2024) though longitudinal studies either found no association (Taniguchi et al., 2025) or reported that caregiving was associated with a decrease in smoking (Hiyoshi et al., 2023). Overall, few studies have examined caregiving transitions or younger caregivers. Only three longitudinal studies, all restricted to samples of older adults aged 50 and above, have investigated the initial transition from non-caregiving into caregiving (Hiyoshi et al., 2023; Taniguchi et al., 2025; Zan & Shin, 2022), and, to our knowledge, no study to date has investigated transitions out of caregiving in relation to health behaviours changes. This study addresses these gaps by examining the longitudinal associations between entering caregiving, caregiving cessation, and changes in health behaviours across the adult lifecourse in the UK and whether these associations differed by sex and lifecourse stage in which caregiving occurs.

Based on the theoretical framework outlined above, we hypothesised that: (H1) transitioning into caregiving would be associated with changes in health behaviours, with the potential for both improvements (e.g. through enhanced health awareness) and deterioration (e.g. through stress-related coping); (H2) exiting caregiving would be associated with a reversion of health behaviours towards pre-caregiving levels; (H3) associations would be more pronounced among higher-intensity caregivers; (H4) women would experience less favourable behavioural changes, given gendered division of unpaid care where women provide more hours of intensive care and more frequently combine it with paid work and domestic duties, leaving less capability and opportunity for health-promoting behaviours (Lacey et al., 2019; Pinquart & Sörensen, 2006); and (H5) associations would be stronger among younger adults, for whom caregiving is less socially normative. Lifecourse theory proposes that a transition's effects depend on its timing and on available resources (Bartley, 2017; Dannefer, 2003), and younger adults may have had fewer opportunities to accumulate resources and may face greater conflict with education and early career (Koumoutzis et al., 2020; Letelier et al., 2026).

## 2 Methods

### 2.1 Study design and data

This study draws on data from the UK Household Longitudinal Study (UKHLS)(University Of Essex, 2023), a nationally representative panel study of households across the UK. Initiated in 2009, the study recruited around 40,000 households using a stratified, clustered probability sampling approach. All individuals aged 16 and over within selected households are invited to participate in annual interviews. The inclusion of participants from age 16 reflects this sampling frame and was retained to capture caregiving across the full adult lifecourse, including young carers whose experiences are increasingly recognised as distinct and policy-relevant (Joseph et al., 2020; Lacey et al., 2022). Attrition patterns in UKHLS are comparable to those observed in other longitudinal studies (Benzeval et al., 2023), and by wave 13 the study had lost 64.5% of its initial sample (Cabrera-Álvarez & Lynn, 2023). Participants provided oral informed consent, and the study received ethical approval from the University of Essex Ethics Committee.

### 2.2 Participants

The present analysis uses data from waves 5 through 13 for analysing smoking, covering the period from 2013 to 2023. For physical inactivity, diet and alcohol consumption wave 7, 9, 11 and 13 (covering period from 2015 to 2023) was used. Wave 12 data for physical activity, diet, and alcohol consumption were excluded because the relevant survey questions were only introduced mid-fieldwork during the COVID-19 pandemic, limiting comparability with other waves. Smoking data from wave 12 were retained as they were collected throughout fieldwork. Participants aged 16 or older were eligible if they completed both the interviewer-administered and self-completion components of the UKHLS survey. Only those contributing at least two observations on caregiving status and health behaviours were included. A total of 49,903 individuals had caregiving and health behaviour data observed across at least one of waves 7, 9, 11, and 13. These waves were selected because the IPAQ and AUDIT-C measures used in this study were only available from wave 7 onwards on a biennial cycle. For the smoking analysis, 61,010 individuals were available, drawing on data from waves 5 to 13. Across both samples, slightly more than half of participants were women (53.5% and 52.9% respectively), and the mean age at baseline was 45.4 years (SD = 19.4) and 43.6 years (SD = 19.5) respectively. After applying inclusion criteria, between 6263 and 35,779 participants were available for analysis depending on the outcome. Detailed participant flow charts are provided in the Supplement 4.

### 2.3 Measures

#### 2.3.1 Dependent variables

Four health behaviours were examined as outcome variables, namely physical inactivity, fruit and vegetable intake, problematic alcohol consumption, and smoking. Physical inactivity was derived as a binary variable based on responses to the short form of the International Physical Activity Questionnaire (IPAQ) (Egeland et al., 2008), using definitions consistent with the physical activity guidelines issued by the UK Chief Medical Officer (Department of Health and Social Care, 2019). The short-form IPAQ, on which the physical activity measure is based, has demonstrated acceptable test-retest reliability but only moderate criterion validity when compared to accelerometer-derived measures, with accuracy varying by demographic group (Craig et al., 2003; Lee et al., 2011; Quinlan et al., 2021). Participants were asked to report the frequency and duration of their engagement in moderate and vigorous physical activity. Individuals were classified as physically active if they reported engaging in at least 75 min of vigorous activity per week, 150 min of moderate activity per week, or a combination of both totalling a minimum of 150 min per week. Those not meeting these thresholds were categorised as physically inactive. While dichotomising physical activity minutes may lead to a loss of information, the thresholds used are well validated against major health outcomes including all-cause mortality and cardiovascular disease (Arem et al., 2015; Bull et al., 2020; Lee et al., 2022).

Dietary behaviour was assessed based on participants’ average daily intake of fruits and vegetables. Participants were asked how many days per week they typically consumed fruit and, on those days, how many portions they usually ate. Equivalent questions were asked regarding vegetable consumption. Using these responses, the total number of fruit and vegetable portions consumed per week was calculated, and a daily average was derived. While self-reported dietary intake is subject to social desirability and recall biases, brief fruit and vegetable instruments have demonstrated acceptable validity for population-level monitoring, with correlations of 0.4 to 0.5 against more intensive dietary assessment methods (Kim & Holowaty, 2003).

Alcohol consumption was assessed using the Alcohol Use Disorders Identification Test–Consumption (AUDIT-C) (Bradley et al., 2003; Bush et al., 1998; Duffy et al., 2023), a validated three-item screening tool. The AUDIT-C measures the frequency of alcohol intake, the typical number of drinks consumed on a drinking day, and the frequency of binge drinking which is defined as consuming more than 8 units of alcohol on a single occasion for men, and more than 6 units for women. Total scores range from 0 to 12, with higher scores indicating more hazardous drinking behaviour. Participants were classified as ‘problematic drinkers’ if they scored ≥3 for females or ≥4 for males, consistent with cut-off points used in previous research (Denham et al., 2020; Hernandez Chilatra et al., 2024; Kearns et al., 2017). The AUDIT-C has shown good sensitivity and specificity for identifying hazardous drinking in population settings (Bradley et al., 2003; Bush et al., 1998).

Smoking status was assessed via a self-reported question asking participants whether they currently smoked. Those who responded “yes” were classified as current smokers, while those who responded “no” were categorised as non-smokers. Self-reported smoking status generally shows high concordance with biochemical validation (Patrick et al., 1994). Information on the number of cigarettes smoked per day was also available; supplementary analyses using two-part models to account for the semi-continuous distribution of these variable produced results consistent with the binary classification (Supplement 10).

#### 2.3.2 Independent variable

In each wave of the survey, participants were asked questions regarding their caregiving status and the intensity of any care provided. They were asked: “Do you provide some regular service or help for any sick, disabled or elderly person living with you?” and, in an equivalent question, whether they “provide some regular service or help for any sick, disabled or elderly person not living with you.” A binary caregiving status variable (yes/no) was derived from these questions. Respondents who reported providing care both within and outside their household were classified as dual caregivers. Caregiving intensity was based on the number of hours of care provided per week which were collected as categorical response bands rather than as a continuous measure. Participants who provided fewer than 20 h of care per week were classified as low-intensity caregivers, and those providing 20 h or more per week were categorised as high-intensity caregivers. The 20-h threshold is commonly used in UK caregiving research and aligns with evidence that health outcomes, including psychological distress and mental health functioning, worsen at this level of caregiving intensity (Hirst, 2002; Lacey et al., 2024).

#### 2.3.3 Covariates

Covariates differed by analytical approach. In the fixed-effect models, no time-varying covariates were adjusted for, as these were considered to lie on the causal pathway between caregiving transition and health behaviours. The fixed-effects specification inherently accounted for all time-invariant confounders (observed and unobserved). In contrast, the piecewise growth curve models were estimated on a propensity score matched sample based on baseline covariates including age groups, sex, ethnicity, household income quintiles, occupational class, employment status, de-facto marital status, highest educational attainment, household size and number of children living in the household, self-rated general health, psychological distress (GHQ-12) and physical health functioning (SF-12). The total number of waves each participant contributed and their baseline wave were also included in the matching to account for differences in follow-up duration. Full variable descriptions can be found in Supplement 1.

### 2.4 Statistical analysis

We employed two complementary approaches, with the fixed effects (FE) models as the primary specification and the piecewise growth curve models on a propensity score matched sample as a complementary robustness check. Both analyses focused on the first observed caregiving transition. The FE models were treated as primary because they control for all time-invariant confounders, observed and unobserved, and estimate the average within-person change associated with caregiving transitions, providing the stronger basis for inference in observational data. Their main limitation is that they rely on within-individual variation, so for binary outcomes only individuals who change state contribute to estimation and precision is reduced where such variation is limited. The piecewise growth curve models served as a complementary robustness check that additionally modelled the timing and shape of behavioural change before, during, and after the transition relative to a matched comparison group, which the FE specification does not capture. For the FE models, we used only within-individual variation over time, which controls for stable characteristics such as genetic predispositions, early-life experiences, and personality traits (Gardiner et al., 2009; Hill et al., 2020). We estimated entry into caregiving and exit from caregiving in separate fixed effects models, each capturing the within-person change in health behaviours associated with that transition. We did not adjust for time-varying covariates, as we considered them to be on the causal pathway between caregiving transitions and the outcomes of interest. This decision was informed by our directed acyclic graph (DAG)(Textor et al., 2016), provided in the Supplement 2, which outlines the hypothesised relationships between caregiving transitions, time-varying factors, and health behaviour outcomes.

Second, to capture the complex temporal dynamics before, during, and after a caregiving transition, we additionally performed piecewise growth curve modelling on a propensity score matched sample following the approach by Lacey and colleagues (Lacey et al., 2024) and employed entropy balancing as a complementary reweighting method to correct for residual covariate imbalance after matching (Cefalu et al., 2020; Hainmueller, 2012; McMullin & Schonberger, 2022; Zhao & Percival, 2017). In these models, the “piecewise” specification allowed separate slopes to be estimated for the period before, during and after the caregiving transition, centred on the caregiving transition (time = 0). Piecewise growth curve models partitioned the health trajectory into three segments: (1) pre-transition, (2) transition year, and (3) post-transition. Interaction terms between caregiving status and slope changes were included to assess whether behavioural changes differed between groups.

Matching was performed at a 1:3 ratio, with each caregiver matched to up to three non-caregivers. Exact matching was applied for sex, age at baseline, and baseline wave to account for period effects. Covariate balance was assessed before and after matching using standardised mean differences. To enable comparable trajectory modelling, each matched control was assigned the same transition time point as their matched caregiver, so that time could be centred around the caregiving transition (time = 0) for both groups. In the piecewise specification, interaction terms between the spline components and the caregiving transition variable were used to test whether the change in slope at the transition point and in the post-transition period differed between caregivers and non-caregivers. A p-value of ≤0.05 for these interaction terms was taken as evidence that the trajectory of health behaviours changed differentially around the caregiving transition. Further technical detail on the matching procedure is provided in Supplement 3. For smoking, which was available across nine waves, trajectories spanned up to seven years before and after the transition. For physical inactivity, problematic drinking, and fruit and vegetable consumption, only four waves were available; therefore, trajectories covered up to four years before and up to six years after caregiving onset. Binary outcomes (physical inactivity, smoking, and problematic drinking) were modelled using average marginal predicted probabilities, while diet (measured as portions of fruit and vegetables per day) was treated as a continuous outcome, with average marginal effects estimated.

Two separate analyses were conducted. The first examined transitions into caregiving, including only individuals who were non-caregivers at baseline. The second focused on caregiving exit. In this case, individuals who stopped caregiving were initially compared to those who continued to provide care. However, due to the limited availability of matched continuous caregivers as most exited caregiving, a secondary comparison was made between those who exited caregiving and those who remained non-caregivers. Analyses were stratified by gender and lifecourse stage, as well as by caregiving hours and place of care. Participants with missing baseline health or covariate data were excluded. Models were estimated using maximum likelihood in Stata 17. For the piecewise growth curve models, the mkspline (Orsini & Greenland, 2011) command in Stata was used to test the difference in slope changed during and after the caregiving transition through interaction terms between each spline component and caregiving transition status. Data from the UKHLS are available to registered researchers via the UK Data Service (https://ukdataservice.ac.uk). The full data cleaning and analysis code used in this study has been deposited at GitHub (https://github.com/EnricoPfeifer/caregiving-transitions-healthbehaviour-uk). A comparison of the covariate balance before and after matching can be found in Supplement 8.

### 2.5 Sensitivity analysis

We carried out three sensitivity analyses. First, to test whether caregiving was associated with smoking intensity and not only smoking status, we modelled the number of cigarettes smoked per day, a heavily right-skewed measure with many non-smokers, using two-part models within the piecewise framework on the matched sample (Supplement 10). Second, because physical inactivity and problematic drinking were derived by dichotomising the IPAQ minutes and the AUDIT-C score, we re-estimated these two outcomes using their continuous, standardised scales in both the fixed effects and piecewise models (Supplement 11); current smoking is binary and diet was already continuous, so this did not apply to them. Third, to assess missing covariate data, we re-estimated the piecewise models under multiple imputation by chained equations, imputing baseline covariates and the baseline outcome, with matching and estimation within each of 10 imputed datasets pooled using Rubin's rules (Supplement 12).

## 3 Results

### 3.1 Descriptive statistics

For the transition into caregiving sample (Table 1), the matched population included 4436 to 4468 individuals for the PA, diet, and problematic drinking, and 8659 for smoking. In contrast, the transition out of caregiving sample comprised between 3340 and 3371 individuals for the PA, diet, and alcohol analyses, and 5385 for smoking. Across both analyses, the gender distribution was skewed towards women (approximately 59–63%), and the ethnic composition was predominantly White (>85%), with smaller representation from Black, South Asian, and other minority ethnic groups. Participants who exited caregiving tended to be older than those entering, with a mean age of 54.5 years versus 50.9 years for PA, diet, and alcohol outcomes, and 49.9 versus 48.0 for smoking. Exit groups also had a higher proportion of individuals in late adulthood (65+), lower educational attainment, and were more likely to be not employed or in lower occupational classes. Exiters also had a higher proportion of individuals not in paid employment and living in smaller households without children. Across transitions, over 70% of participants were married or cohabiting. The majority rated their health as good or better, although those exiting caregiving reported slightly worse physical health (SF12-p) and marginally higher psychological distress (GHQ scores). Further descriptive tables stratified by age group and gender as well as descriptive transitions patterns are in Supplement 5-7.

**Table 1:** **Baseline characteristics of participants who transitioned into or out of unpaid caregiving in the propensity score matched sample.** Sociodemographic and health-related characteristics are shown for the treated group only (i.e., participants who transitioned into or out of caregiving). The corresponding control group is not presented separately because, following propensity score matching and entropy balancing, no statistically significant differences remained between treated and control groups on any observed covariate (all p > 0.05). Summary statistics are shown separately for each health behaviour outcome (physical inactivity, diet [fruit and vegetable consumption], problematic drinking, and smoking) and by transition type (entry into caregiving [Entering] and cessation of caregiving [Exit]). Matching was conducted separately for each outcome using propensity scores that included the corresponding baseline health behaviour. Percentages are shown for categorical variables and means with standard deviations (SD) for continuous variables. Sample sizes vary across outcomes due to outcome-specific matching procedures and wave availability.

Outcome		Physical inactivity		Diet (fruit and vegetable consumption)		Problematic drinking		Smoking	
Caregiving transition		Entering	Exit	Entering	Exit	Entering	Exit	Entering	Exit

**N**		**N=4,436**	**N=3,340**	**N=4,468**	**N=3,363**	**N=4,468**	**N=3,371**	**N=8,659**	**N=5,385**

Sex	Male	40.6%	37.3%	40.6%	37.4%	40.5%	37.4%	41.6%	39.9%
	Female	59.4%	62.7%	59.4%	62.6%	59.5%	62.6%	58.4%	60.1%
Age group	Early-adulthood (16-29)	10.4%	8.6%	10.5%	8.5%	10.5%	8.5%	16.6%	16.1%
	Early mid-adulthood (30-49)	34.8%	26.2%	34.7%	26.1%	34.6%	26.2%	35.6%	29.2%
	Late mid-adulthood (50-64)	33.9%	35.7%	33.9%	35.6%	34.0%	35.6%	28.8%	33.0%
	Late adulthood (65+)	20.9%	29.6%	21.0%	29.7%	20.9%	29.7%	18.9%	21.8%
Education	No Qualification	8.2%	11.3%	8.9%	11.7%	8.9%	11.8%	10.3%	11.6%
	A-level, GCSE	51.3%	52.2%	51.8%	52.6%	51.8%	52.6%	52.3%	54.7%
	Degree or other higher qualification	40.5%	36.5%	39.2%	35.7%	39.3%	35.7%	37.3%	33.6%
Ethnicity	White	86.8%	87.7%	86.8%	87.8%	86.7%	87.7%	85.1%	85.7%
	Black	4.3%	3.1%	4.3%	3.1%	4.3%	3.1%	4.6%	3.4%
	Indian	2.9%	3.3%	2.9%	3.3%	3.0%	3.3%	3.1%	3.1%
	Pakistani/Bangladeshi	3.8%	4.2%	3.7%	4.1%	3.8%	4.2%	4.6%	5.4%
	Other	2.3%	1.7%	2.3%	1.7%	2.3%	1.8%	2.6%	2.4%
Occupational class	Not employed	38.7%	50.8%	38.9%	51.0%	38.9%	51.0%	42.2%	48.5%
	Management & professional	27.8%	19.8%	27.6%	19.7%	27.6%	19.7%	25.0%	20.5%
	Intermediate	14.2%	12.5%	14.1%	12.5%	14.1%	12.5%	13.8%	12.9%
	Routine	19.4%	16.8%	19.4%	16.8%	19.4%	16.8%	19.0%	18.0%
Income quintiles	1 (low)	17.2%	17.1%	17.3%	17.4%	17.3%	17.4%	18.5%	19.2%
	2	20.5%	21.4%	20.5%	21.3%	20.5%	21.3%	20.9%	21.5%
	3	19.5%	21.2%	19.6%	21.1%	19.5%	21.1%	20.2%	20.4%
	4	21.5%	21.5%	21.4%	21.4%	21.4%	21.4%	20.2%	20.1%
	5 (high)	21.4%	18.9%	21.3%	18.9%	21.3%	18.8%	20.1%	18.8%
Employment status	Not in paid employment	36.3%	47.9%	36.4%	48.1%	36.4%	48.1%	39.1%	45.5%
	Full-time employed	45.6%	35.0%	45.6%	34.8%	45.6%	34.9%	43.6%	36.1%
	Part-time employed	18.2%	17.1%	18.0%	17.0%	18.1%	17.0%	17.3%	18.4%
Number of children in the household	0	72.1%	80.1%	72.1%	80.1%	72.2%	80.1%	72.3%	77.8%
	1	11.8%	8.7%	11.8%	8.6%	11.8%	8.7%	11.8%	9.8%
	2	11.8%	8.4%	11.8%	8.4%	11.8%	8.4%	11.1%	8.3%
	3+	4.3%	2.8%	4.3%	2.8%	4.3%	2.9%	4.8%	4.1%
Household size	1	12.7%	13.7%	12.7%	13.9%	12.7%	13.9%	12.5%	11.9%
	2	38.2%	43.5%	38.1%	43.5%	38.1%	43.5%	35.9%	28.9%
	3-4	38.6%	32.3%	38.6%	32.2%	38.5%	32.2%	38.4%	34.4%
	5+	10.6%	10.5%	10.6%	10.4%	10.6%	10.5%	13.2%	14.9%
Cohabiting status	Single, separated, widowed	28.2%	29.1%	28.3%	29.1%	28.2%	29.2%	32.9%	34.3%
	Married or cohabiting	71.8%	70.9%	71.3%	70.9%	71.8%	70.8%	67.1%	65.7%
Self-rated general health	Excellent, very good, good	79.2%	78.3%	79.1%	78.2%	79.2%	78.2%	80.5%	79.4%
	Fair or poor	20.8%	21.7%	20.9%	21.8%	20.8%	21.8%	19.5%	20.6%
Age	Mean (SD)	50.9 (15.6)	54.5 (15.9)	50.9 (15.6)	54.5 (15.9)	50.9 (15.6)	54.4 (15.9)	48.0 (17.0)	49.9 (17.6)

GHQ	Mean (SD)	11.1 (5.6)	11.3 (5.6)	11.1 (5.6)	11.3 (5.6)	11.1 (5.6)	11.3 (5.6)	11.3 (5.9)	11.5 (6.0)

SF12-p	Mean (SD)	49.5 (10.9)	48.7 (11.0)	-	-	-	-	-	-

### 3.2 Fixed effect models

#### 3.2.1 Caregiving onset

Fixed effects models (Table 2) revealed that transitioning into caregiving was associated with significant changes in several health behaviours, with evidence of variation by caregiving intensity and age group. Entering caregiving was associated with 16% lower odds of being physically inactive (OR = 0.84, 95% CI: 0.79/0.89) compared with non-caregivers. This association was consistent across both lower-intensity (<20 h/week) and higher-intensity (≥20 h/week) caregivers, with no evidence of effect modification by sex. However, there was significant variation by age group (p = 0.03). The association was strongest among those aged 65+ (OR = 0.77, 95% CI: 0.68/0.88) and in early mid-adulthood (ages 30–49) (OR = 0.81, 95% CI: 0.73/0.90). Among caregivers in early adulthood (16–29), only high-intensity caregiving was associated with reduced inactivity (OR = 0.66, 95% CI: 0.46/0.95). Regarding daily fruit and vegetable consumption, no statistically significant change was observed overall following entry into caregiving (β = 0.02, 95% CI: −0.01/0.06). There was no evidence of effect modification by sex, age, or caregiving hours in relation to fruit and vegetable consumption. For problematic drinking, entering caregiving was marginally associated with higher odds of problematic alcohol use (OR = 1.09, 95% CI: 1.00/1.19) but only among low-intensity caregivers (<20 h/week) (OR = 1.11, 95% CI: 1.01/1.22). The interaction between caregiving entry and age group for problematic drinking was not statistically significant (p = 0.07). Regarding smoking, caregiving was associated with increased odds of being a smoker (OR = 1.16, 95% CI: 1.07/1.27), for both low- and high-intensity caregiving. There was no evidence of effect modification by sex or age.

**Table 2:** **Fixed effects model estimates for changes in health behaviours following entry into unpaid caregiving.** Estimated effects from fixed effects models examining within-individual changes in four health behaviours (physical inactivity, fruit and vegetable consumption, problematic drinking, and smoking). Results for physical inactivity, problematic drinking, and smoking are reported as odds ratios with 95% confidence intervals in parentheses. The diet outcome (daily fruit and vegetable consumption) is modelled as a continuous variable and reported as beta coefficients with 95% confidence intervals. Models include main effects for caregiving status and caregiving hours, and interaction terms with sex and age group. Separate models were estimated for each outcome; sample sizes vary accordingly. P-values indicate significance of main effects and interaction terms.

	Physical inactivity (n = 18,255)	p-value	Diet (n = 46,363)	p-value	Problematic drinking (n = 9,455)	p-value	Smoking (n = 6,263)	p-value
	OR (95% CI)		β (95% CI)		OR (95% CI)		OR (95% CI)	
Caregiving status	0.84 (0.79/0.89)	<0.001	0.02 (−0.01/0.06)	0.16	1.09 (1.00/1.19)	0.05	1.16 (1.07/1.27)	0.001

Caregiving hours		<0.001		0.54		0.09		<0.001
<20 h/week	0.84 (0.79/0.90)		−0.01 (−0.01/0.00)		1.11 (1.01/1.22)		1.15 (1.05/1.27)	
≥ 20 h/week	0.83 (0.74/0.93)		0.00 (−0.02/0.01)		1.05 (0.89/1.24)		1.17 (1.00/1.37)	

Caregiving status *sex		0.51		0.13		0.20		0.84
Male	0.82 (0.74/0.90)	<0.001	−0.01 (−0.06/0.04)	0.70	1.17 (1.02/1.35)	0.03	1.16 (1.02/1.32)	0.02
Female	0.85 (0.79/0.91)	<0.001	0.04 (0.00/0.09)	0.05	1.04 (0.93/1.17)	0.45	1.16 (1.04/1.30)	0.01

Caregiving status*age group		0.03		0.93		0.13		0.20
Early adulthood (16-29)	0.95 (0.80/1.13)	0.56	0.04 (−0.07/0.15)	0.45	1.12 (0.90/1.40)	0.30	1.09 (0.93/1.29)	0.28
Early mid-adulthood (30-49)	0.81 (0.73/0.90)	<0.001	0.01 (−0.05/0.07)	0.76	1.10 (0.95/1.29)	0.21	1.10 (0.96/1.26)	0.18
Late mid-adulthood (50-64)	0.90 (0.81/0.99)	0.04	0.04 (−0.02/0.10)	0.18	0.94 (0.80/1.09)	0.41	1.22 (1.02/1.46)	0.03
Late adulthood 65+	0.77 (0.68/0.88)	<0.001	0.00 (−0.06/0.07)	0.91	1.20 (0.99/1.46)	0.06	1.36 (1.02/1.83)	0.04

Caregiving hours*sex		0.81		0.19		0.27		0.90
Male		<0.001		0.87		0.03		0.09
<20 h/week	0.83 (0.74/0.91)		0.00 (−0.06/0.05)		1.22 (1.05/1.42)		1.15 (1.00/1.32)	
≥ 20 h/week	0.79 (0.65/0.96)		−0.03 (−0.14/0.08)		1.06 (0.80/1.41)		1.21 (0.94/1.56)	
Female		<0.001		0.06		0.75		0.05
<20 h/week	0.85 (0.78/0.92)		0.03 (−0.02/0.08)		1.05 (0.93/1.18)		1.16 (1.02/1.31)	
≥ 20 h/week	0.85 (0.74/0.98)		0.09 (0.01/0.18)		1.04 (0.84/1.28)		1.13 (0.94/1.37)	

Caregiving hours*age group		0.001		0.07		0.01		0.08
Early adulthood (16-29)		0.07		0.17		0.47		0.38
<20 h/week	1.01 (0.84/1.22)		0.09 (−0.03/0.20)		1.16 (0.92/1.47)		1.05 (0.88/1.25)	
≥ 20 h/week	0.66 (0.46/0.95)		−0.09 (−0.31/0.13)		1.03 (0.65/1.63)		1.25 (0.90/1.72)	

Early mid-adulthood (30-49)		<0.001		0.31		0.09		0.27
<20 h/week	0.78 (0.70/0.87)		−0.01 (−0.08/0.05)		1.08 (0.92/1.28)		1.12 (0.97/1.30)	
≥ 20 h/week	0.98 (0.81/1.19)		0.08 (−0.04/0.20)		1.38 (1.02/1.85)		1.00 (0.79/1.27)	

Late mid-adulthood (50-64)		0.11		0.03		0.38		0.09
<20 h/week	0.89 (0.80/0.99)		0.02 (−0.04/0.08)		0.93 (0.79/1.10)		1.23 (1.01/1.49)	
≥ 20 h/week	0.91 (0.75/1.12)		0.15 (0.04/0.26)		0.82 (0.61/1.11)		1.24 (0.90/1.70)	

Late adulthood 65+		<0.001		0.43		0.01		0.14
<20 h/week	0.82 (0.72/0.95)		0.03 (−0.05/0.10)		1.36 (1.09/1.69)		1.40 (1.00/1.97)	
≥ 20 h/week	0.66 (0.52/0.83)		−0.06 (−0.18/0.06)		0.91 (0.64/1.29)		1.01 (0.61/1.67)	

#### 3.2.2 Caregiving exit

Exiting caregiving (Table 3) was associated with a 9% increase in the odds of being physically inactive (OR = 1.09, 95% CI: 1.01/1.18), suggesting a potential decline in physical activity once caregiving responsibilities end. This association was modified by age group (p = 0.02), with the strongest association observed among adults aged 65 and older (OR = 1.21, 95% CI: 1.04/1.42). No significant differences were found by sex. There was no evidence of change in fruit and vegetable consumption following caregiving exit (β = 0.00, 95% CI: −0.01/0.02). No interactions with sex or age were statistically significant. Likewise, no significant changes were observed in problematic drinking following caregiving exit (OR = 0.96, 95% CI: 0.86/1.08), and stratified results by sex and age showed no statistical differences. Lastly, no change in smoking behaviour was observed after exiting caregiving (OR = 0.96, 95% CI: 0.86/1.07), with no evidence of effect modification by sex or age group.

**Table 3:** **Fixed effects model estimates for changes in health behaviours following cessation of unpaid caregiving.** Estimated effects from fixed effects models examining within-individual changes in four health behaviours (physical inactivity, fruit and vegetable consumption [diet], problematic drinking, and smoking) before and after caregiving cessation. Results for physical inactivity, problematic drinking, and smoking are reported as odds ratios with 95% confidence intervals in parentheses. The diet outcome (daily fruit and vegetable consumption) is modelled as a continuous variable and reported as beta coefficients with 95% confidence intervals. Models include a main effect for caregiving cessation and interaction terms with sex and age group. Separate models were estimated for each outcome; sample sizes vary accordingly. P-values indicate the significance of the main effects and interaction terms. For interactions and variables with more than two categories, global p-values using Wald-test were reported.

	Physical inactivity (n = 18,262)	p-value	Diet (n = 46,363)	p-value	Problematic drinking (n = 9,455)	p-value	Smoking (n = 6,263)	p-value
	OR (95% CI)		β (95% CI)		OR (95% CI)		OR (95% CI)	
Caregiving status	1.09 (1.01/1.18)	0.03	0.00 (−0.01/0.02)	0.41	0.96 (0.86/1.08)	0.53	0.96 (0.86/1.07)	0.49

Caregiving status *sex		0.21		0.25		0.77		0.50
Male	1.11 (0.98/1.26)	0.09	0.00 (−0.02/0.02)	0.90	0.97 (0.81/1.16)	0.74	0.92 (0.78/1.09)	0.31
Female	1.08 (0.98/1.19)	0.12	0.01 (−0.01/0.02)	0.38	0.96 (0.83/1.11)	0.57	1.00 (0.86/1.16)	0.98

Caregiving status*age group		0.02		0.71		0.11		0.38
Early adulthood (16-29)	1.07 (0.85/1.36)	0.56	0.02 (−0.01/0.05)	0.20	0.97 (0.72/1.32)	0.87	0.77 (0.62/0.96)	0.02
Early mid-adulthood (30-49)	1.14 (0.99/1.31)	0.08	0.00 (−0.02/0.02)	0.90	0.98 (0.79/1.21)	0.86	1.02 (0.86/1.22)	0.82
Late mid-adulthood (50-64)	0.99 (0.87/1.13)	0.89	0.01 (−0.01/0.03)	0.39	1.11 (0.91/1.35)	0.30	1.03 (0.82/1.30)	0.77
Late adulthood (65+)	1.21 (1.04/1.42)	0.02	0.00 (−0.02/0.02)	0.90	0.82 (0.64/1.04)	0.10	1.24 (0.87/1.77)	0.24

#### 3.2.3 Robustness check

##### 3.2.3.1 Caregiving onset

As a robustness check, the piecewise growth curve models on the propensity score matched sample were generally consistent with the fixed effects models. Transitioning into caregiving (Fig. 1) was associated with a temporary reduction in physical inactivity, with a noticeable drop at the point of caregiving onset. This effect gradually diminished over the subsequent four years, eventually aligning with levels seen among non-caregivers. The association was consistent across low- and high-intensity caregivers, although high-intensity caregivers began with higher baseline inactivity. Place of care also influenced trajectories as those caring inside or outside the household saw declines in inactivity, while dual caregivers experienced a delayed increase. No significant differences were observed by sex, but older adults (65+) showed the steepest post-transition increase in inactivity after an initial drop (Fig. 3). Regarding fruit and vegetable consumption, there was no significant change in dietary behaviour associated with transitioning into caregiving across all analyses. No statistically significant association was found between entering caregiving and changes in problematic drinking. Patterns were also stable across sex, age, and place of care, with no modifying effects observed. Regarding smoking, entering caregiving was associated with a significant and sustained increase in smoking prevalence. This increase was particularly pronounced among high-intensity caregivers and among those providing care inside the household or in dual care settings. The smoking increase was most notable in early adulthood (aged 16–29) and early mid-adulthood (30-49), with no significant sex differences in trajectory patterns.

**Fig. 1:**
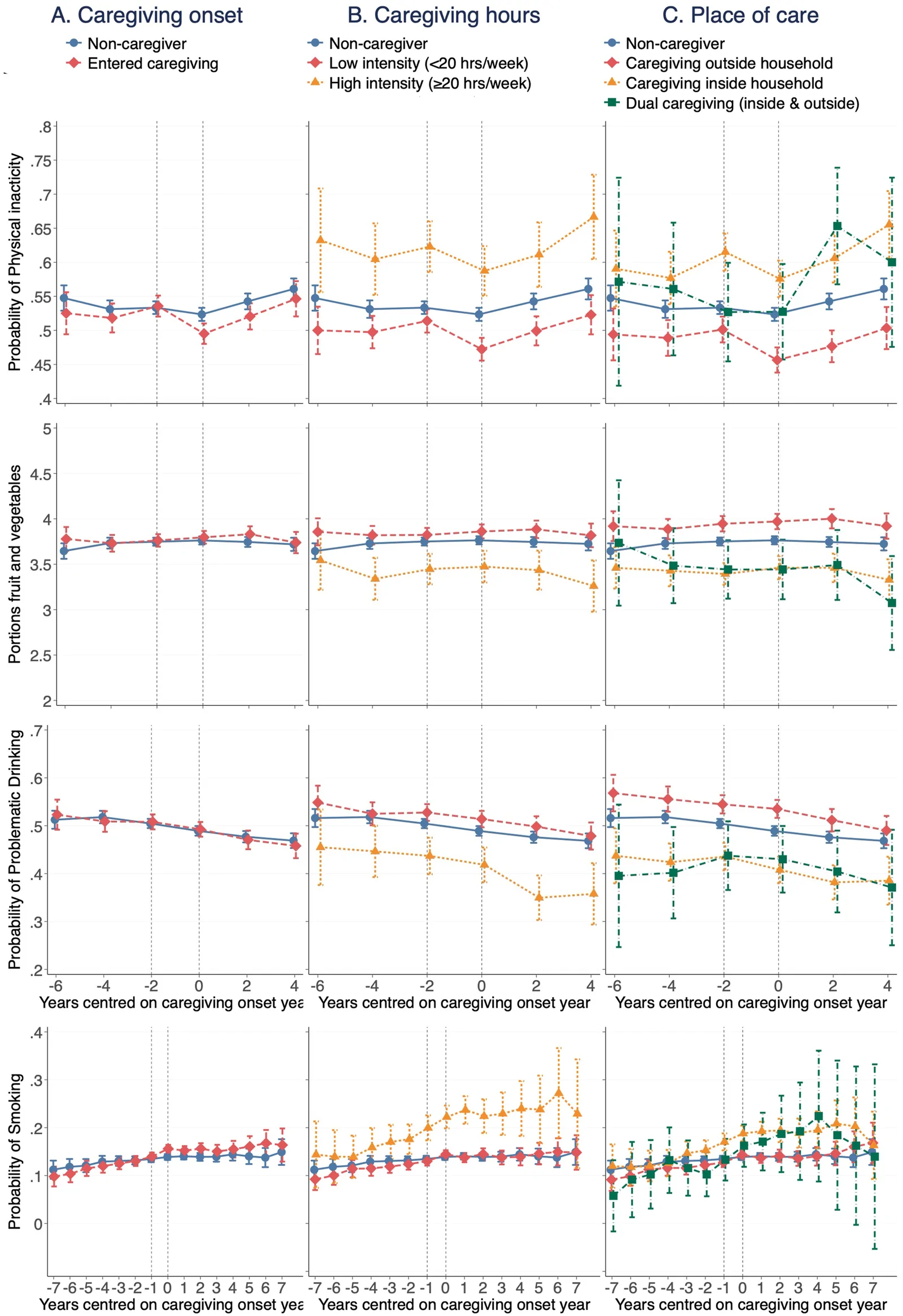
**Predicted health behaviour trajectories before and after entry into unpaid caregiving, stratified by caregiving intensity and place of care.** Results from piecewise growth curve models showing average marginal effects (AMEs) for four health behaviour outcomes: (1) physical activity (top row), (2) fruit and vegetable consumption (second row), (3) problematic alcohol use (third row), and (4) smoking (bottom row). Panel A compares individuals entering caregiving with matched non-caregivers. Panel B stratifies caregivers by intensity (<20 h/week; ≥20 h/week), and Panel C by place of care (outside the household, inside the household, or both). Time is centred on the year of caregiving onset (year 0), with negative values indicating years before and positive values indicating years after the transition. Error bars represent 95% confidence intervals. All models are adjusted for baseline individual characteristics through propensity score matching. Dotted vertical lines delineate the transition to caregiving from −2/-1 year to 0 year.

##### 3.2.3.2 Caregiving exit

Regarding exiting care, piecewise growth curve models were used to analyse changes in health behaviours before and after exiting unpaid caregiving (Fig. 2). These models were centred around the time of caregiving exit and compared to two matched control groups: (1) participants who continued caregiving, and (2) participants who were non-caregivers throughout. Following caregiving exit, the probability of physical inactivity increased among participants who exited caregiving relative to those who continued. This pattern was observed for both men and women. Among women, the divergence was more pronounced, with exited caregivers showing a steady increase in physical inactivity while continued caregivers remained relatively stable. Among men, a similar divergence was observed, though the trajectory of continued caregivers was more variable. No significant interactions were found for place of care or sex overall, although female exiters exhibited a significant increase in inactivity compared to female continuing caregivers (Fig. 4). Older adults also showed more pronounced increases, though age interactions were not statistically significant. Further, exiting caregiving was not associated with significant changes in fruit and vegetable consumption. Trajectories remained stable across all groups, regardless of caregiving intensity, sex, place of care, or age. Although female exiters had slightly lower fruit and vegetable intake in the post-exit period compared to female continuing caregivers (p = 0.01), the magnitude of this association was very small. Findings on problematic drinking yielded no significant association between caregiving exit and problematic drinking. While high-intensity caregivers consistently had lower levels of problematic drinking pre- and post-exit, no significant changes were detected during the caregiving exit. Similarly, no modifying effects were found by sex, place of care, or age group. Smoking patterns following caregiving exit were generally stable, with only minor variations. There was no conclusive evidence of an association between caregiving exit and changes in smoking behaviour when compared to either non-caregivers or continuing caregivers. Among female exiters, a small decline in smoking was observed relative to female continuing caregivers, whereas no meaningful change was found among males. This suggests a potential gender difference in smoking behaviours following caregiving exit. In terms of caregiving intensity and place of care, trajectories differed at baseline but exiting caregiving did not significantly alter smoking behaviour. Notably, exiters in early mid-adulthood (30–49 years) had a statistically significant increase in smoking around and after caregiving exit, though the magnitude of this association was small. For all piecewise regressions, the full table of estimates and p-values per analysis is in Supplement 9.

**Fig. 2:**
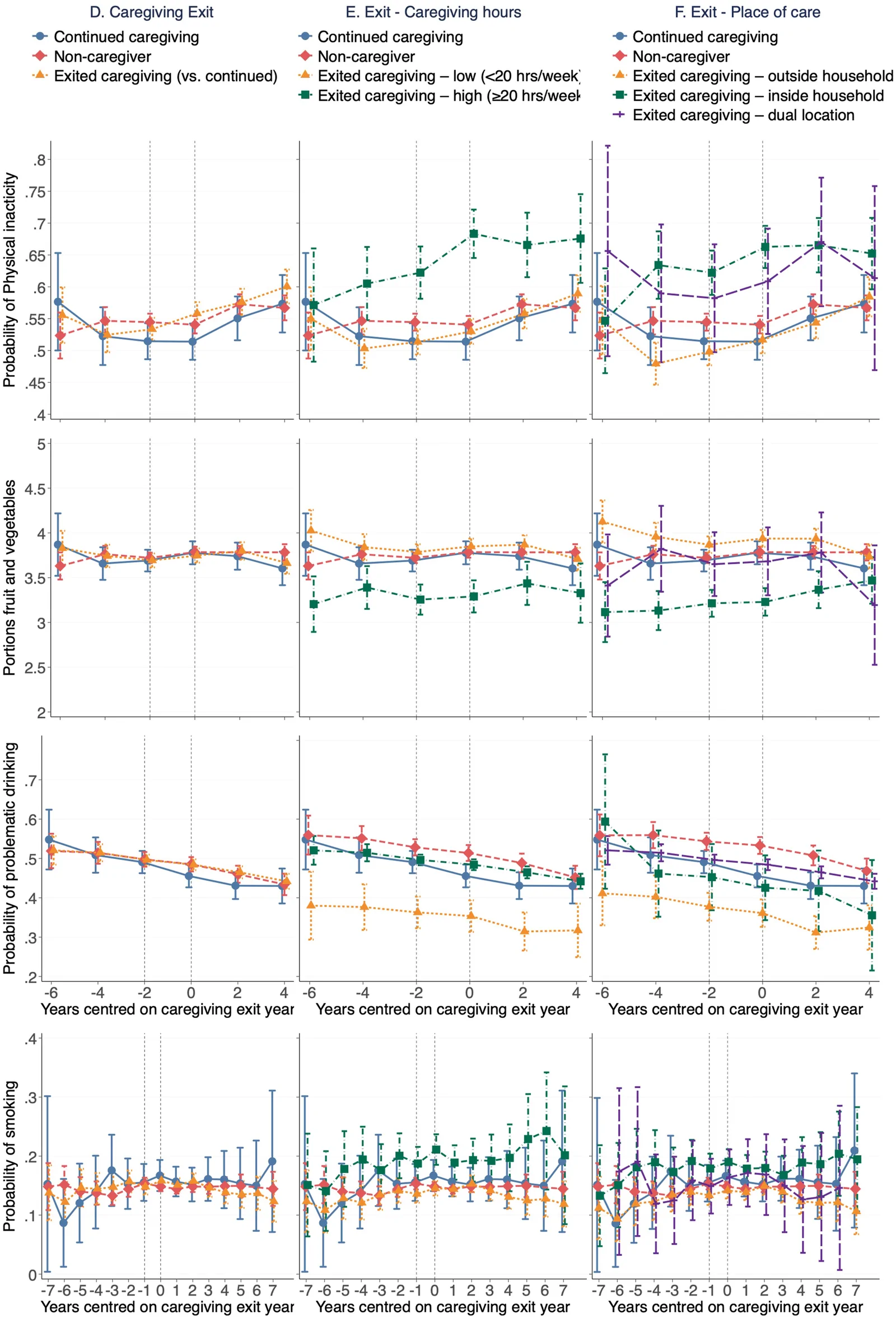
**Predicted health behaviour trajectories before and after cessation of unpaid caregiving, stratified by caregiving intensity and place of care.** Results from piecewise growth curve models showing average marginal effects (AMEs) for four health behaviour outcomes: (1) physical activity (top row), (2) fruit and vegetable consumption (second row), (3) problematic alcohol use (third row), and (4) smoking (bottom row). Each panel presents superimposed results from two separate analyses: (1) comparisons between individuals who exited caregiving and those who continued caregiving, and (2) comparisons between individuals who exited caregiving and matched non-caregivers. Panel D presents results by caregiving status; Panel E by prior caregiving intensity (<20 h/week vs. ≥20 h/week); and Panel F by place of care (outside the household, inside the household, dual location). Time is centred on the year of caregiving cessation (year 0), with negative values indicating years before and positive values indicating years after the transition. Error bars represent 95% confidence intervals. All models are adjusted for baseline individual characteristics through propensity score matching. Dotted vertical lines delineate the transition out of caregiving from −2/-1 year to 0 year.

**Fig. 3:**
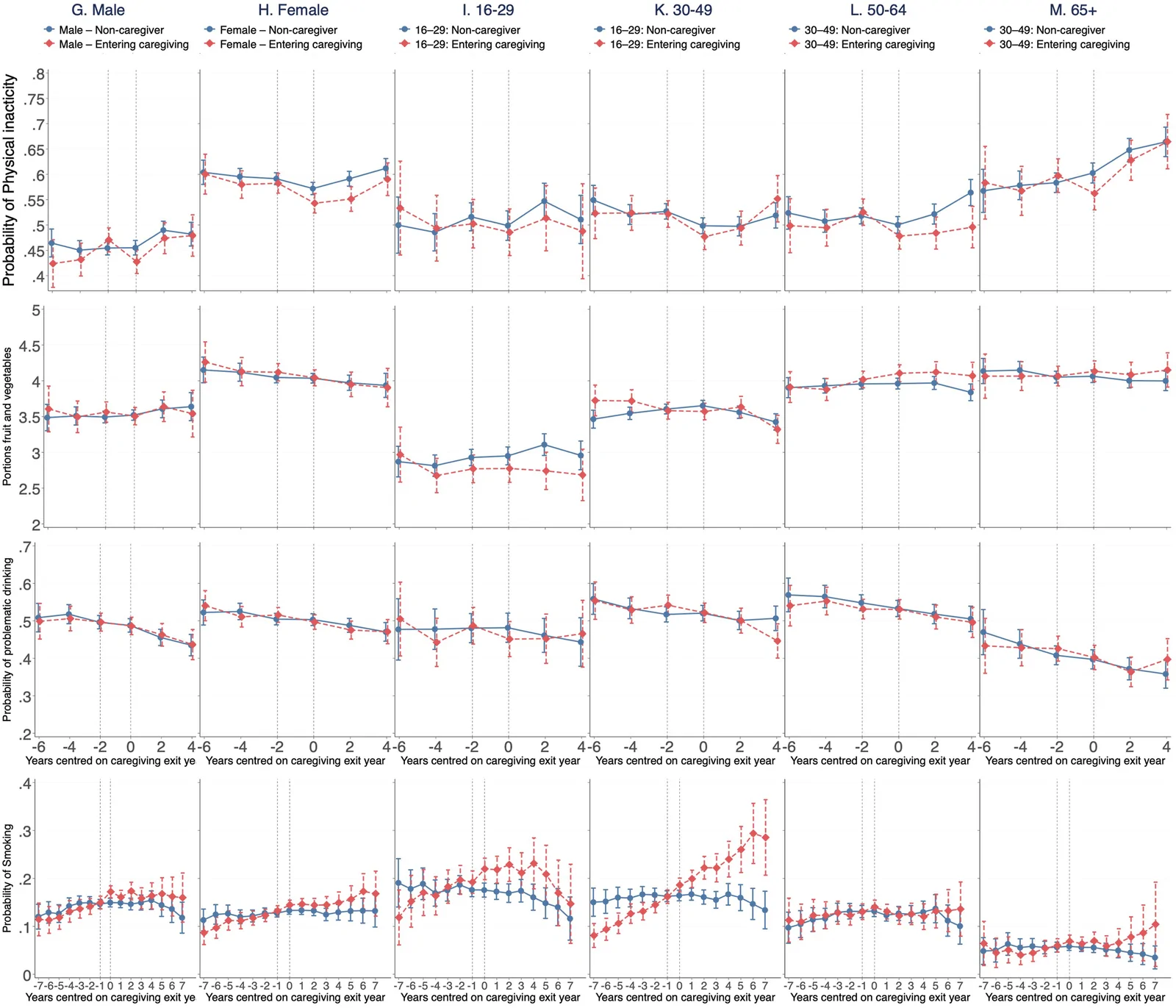
**Predicted health behaviour trajectories before and after entry into unpaid caregiving, stratified by sex and age group. Results** from piecewise growth curve models showing average marginal effects (AMEs) for four health behaviour outcomes: (1) physical activity (top row), (2) fruit and vegetable consumption (second row), (3) problematic alcohol use (third row), and (4) smoking (bottom row). Time is centred on the year of caregiving onset (year 0), with negative values indicating years before and positive values indicating years after transition. Estimates are presented separately for male (G), female (H), and four age groups: 16–29 years (I), 30–49 years (K), 50–64 years (L), and 65+ years (M). Red lines represent caregivers entering caregiving; blue lines represent matched non-caregivers. Error bars represent 95% confidence intervals. All models are adjusted for baseline individual characteristics through propensity score matching. Dotted vertical lines delineate the transition to caregiving from −2/-1 year to 0 year.

**Fig. 4:**
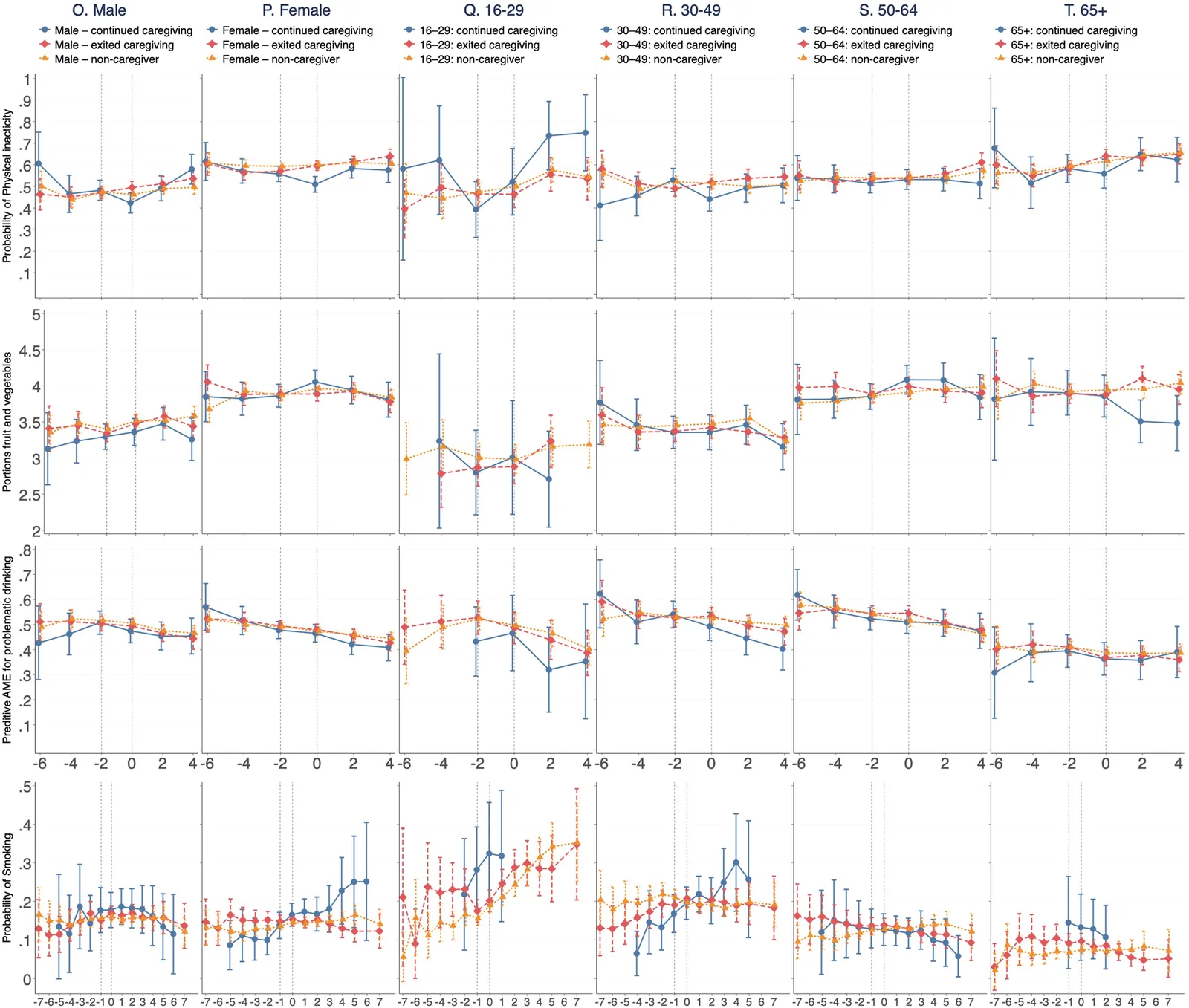
**Predicted health behaviour trajectories before and after cessation of unpaid caregiving, stratified by sex and age group.** Results from piecewise growth curve models showing average marginal effects (AMEs) for four health behaviour outcomes: (1) physical activity (top row), (2) fruit and vegetable consumption (second row), (3) problematic alcohol use (third row), and (4) smoking (bottom row). Each panel presents superimposed results from two separate analyses: (1) comparisons between individuals who exited caregiving and those who continued caregiving, and (2) comparisons between individuals who exited caregiving and matched non-caregivers. Time is centred on the year of caregiving cessation (year 0), with negative values indicating years before and positive values indicating years after the transition. Estimates are presented separately for male (O), female (P), and four age groups: 16–29 years (Q), 30–49 years (R), 50–64 years (S), and 65+ years (T). Error bars represent 95% confidence intervals. All models are adjusted for baseline individual characteristics through propensity score matching. Dotted vertical lines delineate the transition out of caregiving from −2/-1 year to 0 year.

### 3.3 Sensitivity analysis

The sensitivity analyses supported the main findings (Supplements 10 to 12). Modelling the number of cigarettes gave the same picture as the binary smoking analysis: entry into caregiving was associated with more predicted cigarettes smoked in the full sample, but among baseline smokers neither entry nor exit changed consumption, so the association seems to operate through whether people smoke rather than how much. Re-estimating physical inactivity and problematic drinking using their continuous, standardised scales reproduced the main results. Entry into caregiving was associated with a small increase in moderate to vigorous physical activity (β = 0.07; 95% CI: 0.05/0.09), and the decline at exit was in the same direction but weaker, reaching significance only in the piecewise comparison with non-caregivers. The continuous AUDIT-C results were consistent with the main analysis. They showed that entering caregiving was associated with a small increase in the standardised AUDIT-C score in the fixed effects model (β = 0.02, 95% CI: 0.01/0.03), but not in the piecewise growth curve model. This mirrors the binary analysis, where the weak entry association was apparent in the fixed effects but not the piecewise models. Multiple imputation produced results essentially unchanged from the complete-case analysis.

## 4 Discussion

Overall, caregiving transitions were associated with both beneficial and adverse changes in health behaviours. Entering caregiving was linked to an increase in physical activity but also to an increased likelihood of smoking, whereas exiting caregiving was associated with a decline in physical activity without corresponding improvements in other behaviours. While several associations reached statistical significance, the magnitude of the associations was small given the fairly large sample size. The use of binary outcome measures limits the ability to quantify these changes on their original scales. However, these effect sizes are comparable to those reported in other longitudinal studies of caregiving and health behaviours (Hiyoshi et al., 2023; Taniguchi et al., 2025) and may carry public health relevance at the population level given the large number of unpaid caregivers in the UK. These associations were further differentiated by caregiving intensity, with patterns varying across outcomes and between low- and high-intensity caregivers, suggesting that caregivers are not a homogeneous group and that the relationship between caregiving and health behaviours is shaped by the level of care provided.

The finding that entering caregiving increases physical activity is consistent with previous research suggesting that caregivers are more physically active compared to non-caregivers (Fredman et al., 2006; Garcia-Mayor et al., 2021; Gottschalk et al., 2020; Jacob et al., 2020; Kilmer et al., 2024; Nedim et al., 2023; Reeves et al., 2012; Zan & Shin, 2022). However, these findings should be interpreted with caution. Because physical activity was measured as a binary variable based on whether participants met the threshold based on physical activity guidelines, it is not possible to determine whether the observed change reflects genuine increases in health-enhancing leisure-time activity or whether physically demanding caregiving tasks (such as manual handling or assisting with mobility) pushed individuals above the threshold. The IPAQ does not differentiate between occupational and leisure-time physical activity, and it has been noted that occupational physical activity has been linked to adverse health outcomes, including cardiovascular disease and disability (Coenen et al., 2018; Gupta et al., 2020; Holtermann et al., 2018). From a theoretical perspective, these findings can be interpreted through caregiving role theory (Bruhn & Rebach, 2014; Montgomery et al., 2007) where caregiving may generate role strain that constrains leisure-time physical activity while simultaneously introducing physically demanding tasks that mimic moderate activity on self-report measures. Future studies using continuous or accelerometer-based measures of physical activity are needed to clarify whether changes in physical activity during caregiving transitions confer health benefits (Anderson & Durstine, 2019; Durstine et al., 2013; Mahindru et al., 2023; Reiner et al., 2013).

The observed positive association between entry into caregiving and increased likelihood of smoking is consistent with findings from a longitudinal study conducted in Japan by Taniguchi et al. (2025) while a longitudinal analysis of the SHARE study by Hiyoshi et al. (2023) reported decreased odds of smoking. However, direct comparison with both studies is limited, as they were restricted to older adults aged 50 and above, an age group in which we also found no significant association between caregiving and smoking. In the present study, stronger associations were observed among younger adults, particularly those aged 16–29 and individuals in early mid-adulthood (30-49), suggesting that age moderates the relationship between caregiving and smoking behaviour. From a theoretical perspective, these findings can be interpreted through the stress and coping framework and the COM-B model of health behaviour (Michie et al., 2011). Caregiving-related role strain may reduce caregivers' capability and opportunity to engage in healthier coping strategies, while simultaneously increasing motivation to seek immediate stress relief through smoking (Litzelman et al., 2018; Snyder et al., 2014; Teixeira et al., 2018). That the association was strongest among younger caregivers (aged 16-49) is consistent with lifecourse theory, as caregiving at this stage competes with other normative transitions such as education, career development, and family formation, potentially amplifying role strain (Clawson et al., 2016; Slopen et al., 2013).

Findings on problematic drinking were mixed and varied by age, caregiving intensity, and analytic approach. Fixed effects models showed increased odds of problematic drinking after caregiving onset in mid- and later adulthood, but not in younger age groups. In contrast, piecewise growth models found no significant overall association. Although high-intensity caregivers (≥20 h/week) consistently showed lower levels of problematic drinking before, during and after entering caregiving compared to matched controls, this was not associated with transition into caregiving. Discrepancies between the two approaches likely reflect differences in the effective sample and the different estimands. While this means that the evidence on caregiving transitions and problematic drinking remains inconclusive, our study consistently observed a lower probability of problematic drinking among high-intensity caregivers compared to non-caregivers and low-intensity caregivers. However, this pattern was not related to the caregiving transition itself and may reflect health behaviours that precede caregiving, consistent with the 'healthy carer hypothesis' (Bertrand et al., 2012; Fredman et al., 2015).

Regarding diet, no significant associations were found regarding fruit and vegetable consumption. The only comparable longitudinal population-based study, based on the SHARE survey of adults aged 50 and above, found that non-daily fruit and vegetable intake increased among men who became caregivers (Hiyoshi et al., 2023), although differences in population age range and dietary measurement limit direct comparison. Several cross-sectional studies reported lower fruit and vegetable consumption among caregivers (Bailey et al., 2019; Castro et al., 2007; Denham et al., 2020; Savela et al., 2023), while others found higher intake among low-intensity caregivers (Fuchs et al., 2023; Garcia-Mayor et al., 2021). However, one weakness in measuring diet through fruit and vegetable consumption is caregiver's diet might be influenced by stress, time constraints and financial pressures (Dallman et al., 2003; Frey et al., 2024; Petrillo, Valdenegro, et al., 2024). Therefore, fruit and vegetable consumption alone may not fully capture the dietary impact of caregiving which does not reflect stress-related eating behaviours like increased fast food or comfort eating.

Taken together, these findings contribute to theoretical understanding of caregiving and health behaviours in three ways. First, they suggest that caregiving does not operate through a single uniform mechanism on health behaviours; instead, different behaviours are shaped by different combinations of role strain, role enrichment, stress coping, and selection. Second, they provide empirical support for lifecourse-sensitive theoretical models, showing that the behavioural consequences of caregiving vary by the life stage in which caregiving occurs. Third, they point to the need for theoretical frameworks that distinguish between caregiving intensity levels, as low- and high-intensity caregiving appear to follow distinct pathways.

### 4.1 Implications

These findings suggest that caregiving onset is associated with changes in specific health behaviours, particularly physical activity and smoking. The association between caregiving and increased smoking, especially among younger adults (aged 16-49), provides the most direct basis for intervention. Smoking cessation support could be integrated into existing caregiver support programmes, with particular attention to younger caregivers who may be more vulnerable to stress-related smoking. That smoking behaviours did not improve following caregiving exit suggests that cessation support should extend beyond the caregiving period. The observed reduction in physical inactivity is more difficult to translate into recommendations given the inability to distinguish between leisure and caregiving-related activity in the available data. More broadly, our findings indicate that caregiving transitions represent periods of behavioural change, which may inform the timing of health promotion efforts. However, the modest effect sizes observed in this study suggest that individual-level behavioural interventions alone are unlikely to be sufficient. Structural supports such as respite services, flexible working arrangements, and accessible formal care, which are supported by wider evidence on caregiving and health (Brimblecombe & Farias, 2022; Lacey et al., 2024; Xue et al., 2025) may help create conditions in which caregivers can sustain positive health behaviours.

A key priority for future research is to clarify the nature of physical activity changes during caregiving transitions. Longitudinal studies that incorporate accelerometer-derived measures or domain-specific physical activity questionnaires would help determine whether such changes reflect health-enhancing activity or physically demanding caregiving tasks. For smoking, qualitative research with younger caregivers could illuminate the specific stressors and coping pathways that make this group particularly vulnerable and inform the design of targeted cessation interventions. For alcohol consumption, future research should investigate why high-intensity caregivers consistently show lower levels of problematic drinking and whether this reflects selection into caregiving, reduced opportunity for drinking, or other mechanisms not captured in the present study.

### 4.2 Strengths and limitations

Several limitations of this study should be acknowledged. First, all behavioural outcomes were self-reported, which may introduce recall and social desirability biases. Second, the physical activity measure may not distinguish occupational and leisure activity, complicating interpretation. Third, while transitions in caregiving were carefully modelled, there was a two-year gap between observation for most outcomes. Fourth, although the fixed effects models adjusted for wave to account for period effects and the propensity score matching included the baseline wave to ensure temporal comparability, we cannot fully rule out the possibility that the COVID-19 pandemic differentially affected caregivers and non-caregivers in the later waves of the study. Fifth, participants with missing baseline health or covariate data were excluded, which may have introduced selection bias, although multiple imputation on the matched sample yielded consistent results. Sixth, while the dichotomisation of physical activity and problematic drinking was based on validated scales and established cut-off points (Arem et al., 2015; Bull et al., 2020; Department of Health and Social Care, 2019; Saunders et al., 1993), this approach may entail a loss of information and obscure more gradual changes that do not cross the respective thresholds, although sensitivity analysis using continuous measures yielded consistent results. Lastly, while the analysis accounted for observed confounders through propensity score matching and fixed-effects modelling, residual confounding cannot be entirely ruled out. It should also be noted that mechanisms linking caregiving to smoking and drinking may differ for participants aged 16–17, for whom access to tobacco and alcohol is legally restricted, and health behaviour patterns may be more influenced by household and peer environments than individual coping strategies (Loke & Mak, 2013; Millett et al., 2011). However, the small number of participants below age 18 limits the scope for separate analysis of this subgroup. Despite these limitations, this study has several strengths. Most notably, it distinguishes between caregiving entry and exit, offering a dynamic understanding of how transitions into and out of caregiving shape health behaviours over time. Our primary specification, the fixed effects models, controls for all time-invariant confounders, observed and unobserved, and estimates within-person change, providing a strong basis for inference in observational data. The piecewise growth curve models on a propensity score matched sample served as a complementary robustness check, capturing the timing and trajectory of behavioural change around the caregiving transition. The consistency of the direct effects across the two approaches, rarely applied together in research on caregiving and health behaviours, strengthens confidence in the findings. Furthermore, the study's lifecourse focus advances understanding of how caregiving affects health behaviours in early and mid-adulthood, a stage often overlooked in the literature. Nonetheless, as with all observational research, causality cannot be assumed.

## 5 Conclusion

In conclusion, this study provides evidence that caregiving entry and exit shape health behaviours in distinct ways. It underscores the importance of early support for novice caregivers and suggests that some behaviours adopted during the caregiving period may persist beyond the caregiving role.

## CRediT authorship contribution statement

**Enrico Pfeifer:** Writing – original draft, Methodology, Funding acquisition, Formal analysis, Data curation, Conceptualization. **Rebecca Lacey:** Writing – review & editing, Supervision, Methodology, Funding acquisition, Data curation. **Baowen Xue:** Writing – review & editing, Supervision, Methodology, Funding acquisition, Conceptualization. **Hynek Pikhart:** Writing – review & editing, Supervision, Methodology, Conceptualization. **Anne McMunn:** Writing – review & editing, Supervision, Methodology, Funding acquisition, Conceptualization.

## Ethical approval

The project uses secondary data publicly available from the UK Data Service (https://ukdataservice.ac.uk) and Ethical approval for the Understanding Society main study and Innovation Panel was granted by the University of Essex Ethics Committee. Therefore, ethical approval for this project was not required.

## Declaration of generative AI and AI-assisted technologies in the manuscript preparation process

During the preparation of this work, the lead author used Microsoft Copilot in order to assist with language refinement, clarity and grammar. After using this tool, the author(s) reviewed and edited the content as needed and take full responsibility for the content of the published article.

## Funding

This project is funded by the UK Economic and Social Research Council (ESRC) through the UBEL-DTP (UCL, Bloomsbury and East London Doctoral Training Partnership) and is registered under the grant reference ES/P000592/1. The funder was not involved in project design, analysis or write up of findings. AM and BX were supported by funding through the Joint Programming Initiative More Years, Better Lives (JPI MYBL) from the UK Economic and Social Research Council (ES/W001454/1). AM, BX and EP are supported by UK Economic and Social Research Council funding for *Equalise*: ESRC Centre for Lifecourse Health Equity (ES/Z504270/1).

## Declaration of competing interests

The authors declare no competing interests.

## Data Availability

This study uses data from the UK Household Longitudinal Study (UKHLS), which is publicly available through the UK Data Service (https://ukdataservice.ac.uk) under standard access conditions. The analytical code used for this study is available on GitHub (https://github.com/EnricoPfeifer/caregiving-transitions-healthbehaviour-uk).
