# Supplementary material for: Health behaviour changes around transitions into and out of unpaid caregiving in the UK: A longitudinal study
Source: SSM Popul Health. 2026 Sep 21;36:101975. doi: 10.1016/j.ssmph.2026.101975 (PMC13634455; doi:10.1016/j.ssmph.2026.101975)
Supplement: Supplementary file 1 [file mmc1.docx]

Table of Content for Supplement

[S1: Variable definition	2](#_Toc233098656)

[S2: DAG	3](#_Toc233098657)

[S3: Steps for propensity score matching and piecewise growth curve modelling	5](#_Toc233098658)

[S4: Sample Size flow charts	11](#_Toc233098659)

[S5: Descriptive Tables	23](#_Toc233098660)

[S6: Descriptive Outcome by Wave	27](#_Toc233098661)

[S7: Distribution of caregiving transitions by outcomes	28](#_Toc233098662)

[S8: Covariate Balance	29](#_Toc233098663)

[S9: Coefficients and p-values for piecewise regression	31](#_Toc233098664)

[S10: Modelling the Number of Cigarettes	40](#_Toc233098665)

[Distribution of number of cigarettes	40](#_Toc233098666)

[Modelling of the number of cigarettes	41](#_Toc233098667)

[Two-part model vs. poisson regression techniques for numbers of cigarettes smoked	42](#_Toc233098668)

[Caregiving Entry	43](#_Toc233098669)

[Caregiving Exit	45](#_Toc233098670)

[S11: Sensitivity analyses using continuous and standardised outcomes	47](#_Toc233098671)

[S12: Multiple imputation sensitivity analysis	50](#_Toc233098672)

[Reference List for Supplement	55](#_Toc233098673)

# S1: Variable definition

**Table S.1: Variable description**

| **Variable** | **Definition** |
| --- | --- |
| **Physical Activity (PA)** | Binary variable derived from questions on vigorous and moderate activity in the past 7 days, aligned with IPAQ guidelines. Participants classified as physically active (= 0) if they engaged in ≥75 mins vigorous or ≥150 mins moderate PA per week, or 150 mins combined; otherwise coded as physically inactive (= 1). Walking excluded due to question design. |
| **Diet** | Continuous variable based on daily average portions of fruit and vegetables, calculated from frequency and portion size questions. Not dichotomised due to near-normal distribution and lack of consensus on cut-offs. |
| **Smoking** | Binary variable based on response to smoking status question. Participants classified as current smokers (= 1) or non-smokers (= 0). |
| **Alcohol Consumption** | Binary variable for problematic drinking derived from AUDIT-C (3 items). Problematic drinking (=1) defined as score ≥3 (females) or ≥4 (males); otherwise no problematic drinking (=0). |
| **Caregiving Residential Status** | Categorical variable based on responses about caring for household and/or non-household members. Classified as caregiving outside household, inside household or dual (inside and outside household) |
| **Caregiving Status** | Binary variable indicating whether participant is a caregiver or not. |
| **Caregiving Hours** | Categorical variable based on reported weekly hours spent caregiving. Original categories recoded depending on analysis needs. |
| **Sex** | Binary variable: male or female. |
| **Age / Age Groups** | Continuous variable available. Age also grouped into 16–29, 30–49, 50–64, 65+ to reflect life course stages. |
| **Ethnicity** | Categorical variable: white, black, Indian, Pakistani/Bangladeshi, other Asian/other ethnicity. |
| **Relationship Status** | Binary variable: cohabiting (married/partnered) or non-cohabiting (single, divorced, separated, widowed). |
| **Household Size** | Categorical variable: 1-person, 2-person, 3–4 person, 5+ person household. |
| **Number of Children** | Categorical variable: 0, 1, 2, 3+ children under age 16 living in household. |
| **Education** | Categorical variable based on highest qualification: no qualifications, GCSEs/A-Levels/other, or degree/higher. |
| **Employment Status** | Categorical variable: full-time (≥30 hrs), part-time (<30 hrs), not in paid employment. |
| **Occupational Class** | Categorical variable (NS-SEC): managerial/professional, intermediate, routine/manual, or not employed. |
| **Household Income Quintiles** | Categorical variable derived from net household income adjusted by OECD equivalence scale. |
| **Self-rated Health** | Binary variable based on general health rating. Poor/fair = 1, good/very good/excellent = 0. |
| **GHQ-12** | Continuous variable (0–36) from 12-item scale measuring psychological distress. Higher scores indicate more distress. |
| **SF12-PCS** | Continuous variable (0–100) measuring physical health functioning. Higher scores indicate better physical functioning. |
| **Baseline wave** | Wave in which participant was observed first. For Physical activity, diet and problematic drinking wave 7, 9, 11 or 13; and for smoking wave 5 to wave 13. |
| **Number of waves** | Number of waves participants were observed. Range for Physical activity, diet and problematic drinking: 2-4; and for smoking 2-9. |

# S2: DAG

**Figure S.1: Directed Acyclic Graph (DAG)** illustrating assumed causal relationships between caregiving transitions and subsequent health behaviours. This DAG represents the hypothesised relationships between transitioning into or out of unpaid caregiving (exposure) and subsequent health behaviours (outcome). Time-varying covariates (measured at baseline [time A] and follow-up [time B]) include self-rated health, GHQ-12 score, income, occupational class, cohabitation status, and household size. Time-invariant covariates include sex, age, ethnicity, and education. Health behaviour at time A is included to account for baseline behaviour. Minimal sufficient adjustment for estimating the total causal effect of caregiving transition on health behaviour at time B includes: baseline values of cohabitation, GHQ-12, income, occupational class, household size, self-rated health, and health behaviour, along with time-invariant variables (age, sex, ethnicity, and education). Green arrows represent causal paths, pink lines represent open biasing paths, and black arrows indicate variables classified as time-varying. This model was created in DAGitty (Textor et al., 2016).

# S3: Steps for propensity score matching and piecewise growth curve modelling

#### Propensity Score Matching

Propensity Score Matching (PSM) is used in this study to reduce caregiver selection bias. Entry into unpaid caregiving is not random; rather, it is influenced by a range of sociodemographic and contextual factors that make some individuals more likely to transition into a caregiving role than others(Bruhn and Rebach, 2014). PSM addresses this by estimating the probability (propensity score) of becoming a caregiver based on observed baseline characteristics, such as age, gender, employment status, and health. Caregivers are then matched with non-caregivers who have similar propensity scores, helping to balance the distribution of these confounding variables between the two groups. The matched sample is subsequently used in the piecewise growth curve models to compare changes in health behaviours between those who transitioned into caregiving and those who did not.

To enable propensity score matching, a binary treatment variable was created to distinguish participants who transitioned into or out of unpaid caregiving from those who did not. Depending on the study objective, participants were assigned to either the treatment group (“1” = transitioned into or out of caregiving) or the control group (“0” = no caregiving transition). Matching was performed at a 1:3 ratio, with each participant in the treatment group matched to up to three participants in the control group.

Participants were matched on a range of baseline characteristics, including socioeconomic factors (occupational class, household income, highest educational attainment, working status), demographic characteristics (household size, ethnicity, number of children living in the household), health indicators (psychological distress, self-rated health, baseline health behaviour of interest), and the number of waves they had participated in the study and the number of the wave which was the baseline wave. Exact matching was applied for sex, age at baseline, and the wave at which participants entered the study. Matching on the baseline wave was used to account for potential period effects. That is, changes in population-level health behaviours over time, for example declining smoking rates. This ensured that caregivers and non-caregivers were compared within the same temporal context. To assess the quality of the matching, balance diagnostics were conducted using statistical tests (e.g. t-tests and chi-squared tests) and standardised mean differences to ensure covariates were adequately balanced between the treatment and control groups.

#### Piece-wise growth curve models

To achieve the objectives of observing potential changes in health behaviours in response to transitions into caregiving, piecewise growth curve models are considered a viable method to answer the research questions around transition. Piecewise growth curve modelling is a statistical modelling technique that allows to set the knot points or intercept at the beginning of a caregiving period, allowing to assess if and how trajectories differ when study participants enter caregiving. Hence, this is a suitable method to study transitional periods and changes over time (Chou et al., 2004) and can be applied to meet objective 1 and 2 (transitioning in-and out of caregiving).

To test the statistical significance of changes in trajectories before, during, and after the a caregiving transition, the Stata package mkspline was used (Orsini and Greenland, 2011). Three knot points were specified to divide the trajectories into distinct periods or ‘pieces’: the first piece captured the pre-transition trajectory; the second piece captured the transition period which was defined as the interval between the wave before caregiving was first reported and the wave it was first reported; and the third piece captured the post-transition trajectory. Additionally, the option ‘marginal’ was specified to estimate the change in slope from the preceding interval.

These spline components were then included in the regression model through an interaction term between the spline variables and the caregiving transition variable. A p-value of ≤ 0.05 for the interaction term was taken as evidence to reject the null hypothesis of no difference in trajectories across the caregiving transition. Each analysis yielded three p-values: (1) one for the pre-transition segment; (2) one for the transition segment; and (3) one for the post-transition segment, corresponding to the difference in slope between caregivers and non-caregivers in each respective period.

This approach was preferred because piecewise growth curve models allow for the comparison of changes in trajectories across distinct time periods such as before, during, and after the transition into caregiving, rather than assuming a single, continuous trajectory over time. This structure makes it possible to isolate and test whether the slope of health behaviours changes specifically around the caregiving transition. Additionally, testing differences across the entire growth curves may obscure meaningful variation across these time segments, especially given that, due to matching, caregivers and non-caregivers are expected to have similar trajectories prior to the transition.

#### Preparatory steps for piecewise growth-curve models

The time of care transition was observed in those who experienced a caregiving transition which is a crucial variable to model piece-wise growth curves. However, the methodological challenge was the time of caregiving transition could not be observed in the controls because they did not experience a caregiving transition. To be able to model piecewise-growth curves for the non-transitioning control group, the “time of caregiving transition” had to be determined. This was possible by assigning the “time of caregiving transition” for participants without care transition by assigning them the time of transition of their matches who transitioned. This involved several steps.

First, for participants who experienced a caregiving transition, the sample was restricted to participants who had at least one observation prior and after the caregiving transition. Then propensity score matching was conducted as described above. Additionally, unique identifier for each control unit (no caregiving transition) matched to a treatment unit (caregiving transition) was generated. The next step involved managing the matched data to ensure that each treatment case is aligned with the time of caregiving transition from the matched controls. These steps are repeated for each individual in the control group, generating a new variable for the “time of caregiving transition” for each matched control group. The logic ensures that each treatment subject's “time of caregiving transition” is matched with the first occurring “time of caregiving transition” among the matched controls. This process aligns the timing of caregiving transition between the treated and control individuals. Following this, the final analytic sample was selected based on data availability. To be included, participants in both the treatment and control groups needed to have valid observations both before and after the transition period. Therefore, the inclusion criteria were reapplied to ensure that only participants with sufficient data coverage across the pre- and post-transition periods were retained for analysis.

Because some participants in the control group were dropped from analysis after propensity score matching, the sample became slightly imbalanced in view of their distribution of covariates. To adjust for this imbalance, entropy balancing was conducted using the same variables that were used in the propensity score models.

#### Entropy balancing

While PSM is an increasingly popular method in observational studies, it may be difficult to identify a PSM model with an adequate covariate balance (Imai and Ratkovic, 2014). To assess and address potential covariate imbalance in the analytical sample, covariate balance was evaluated both before and after matching using standard statistical test such as t-test and chi-square test. Imbalance was considered present if covariates were statistically different between participants who transitioned (treatment group) and those who did not (control group). Statistical difference was assessed using hypothesis testing (e.g. t-tests or chi-squared tests), and a p-value of 0.05 or smaller was taken to indicate a statistically significant difference between the groups. In the propensity score matched sample, several covariates showed evidence of imbalance, such as baseline health behaviour, ethnicity, number of people living in the household, education and income quintiles.

To tackle the issue of potential covariate imbalance in the analytical sample, Entropy balancing will be used in conjunction with propensity score matching in this study. While propensity score matching estimates the probability of “receiving treatment”, entropy balancing reweights control sample observations to align them with the treated sample in terms of observable covariates (Hainmueller, 2012). Entropy balancing has shown promise in estimating treatment effects especially in scenarios involving a binary exposure, outperforming methods solely focused on propensity score estimation(Zhao and Percival, 2017). Entropy balancing has been found to significantly improve covariate balance in difference-in-difference models(Cefalu et al., 2020). Additionally, entropy balancing has been shown to provide superior covariate balance compared to traditional propensity score weighting models making it a valuable tool inn observational studies where achieving balance between treatment and control group is crucial (McMullin and Schonberger, 2022).

# S4: Sample Size flow charts

Sample sizes

**Figure S.2** Sample size flow chart for physical inactivity using FE models

**Fixed effect models**

Fruit and vegetable consumption

**Figure S.3** Sample size flow chart for fruit and vegetable consumption using fixed effect models.

Drinking

**Figure S.4** Sample size flow chart for problematic drinking using fixed effect models.

Smoking

**Figure S.5** Sample size flow chart for smoking using fixed effect models.

**Figure S.6** Sample size flow chart for propensity score matching and physical inactivity. Only participants in the treatment group with complete baseline covariate and health behaviour data were eligible for propensity score matching. Control group participants were drawn from the pool of non-transitioning individuals with complete baseline data on the same variables.

**Figure S.7** Sample size flow chart for propensity score matching for fruit and vegetables consumption. Only participants in the treatment group with complete baseline covariate and health behaviour data were eligible for propensity score matching. Control group participants were drawn from the pool of non-transitioning individuals with complete baseline data on the same variables.

Drinking

**Figure S.8** Sample size flow chart for propensity score matching and problematic drinking. Only participants in the treatment group with complete baseline covariate and health behaviour data were eligible for propensity score matching. Control group participants were drawn from the pool of non-transitioning individuals with complete baseline data on the same variables.

Smoking

**Figure S.9** Sample size flow chart for propensity score matching and smoking. Only participants in the treatment group with complete baseline covariate and health behaviour data were eligible for propensity score matching. Control group participants were drawn from the pool of non-transitioning individuals with complete baseline data on the same variables.

**Figure S.10** Sample size flow chart for smoking and caregiving exit, comparing exit vs non-caregivers and exit vs long-term caregivers. Only participants in the treatment group with complete baseline covariate and health behaviour data were eligible for propensity score matching. Control group participants were drawn from the pool of non-transitioning individuals with complete baseline data on the same variables.

**Figure S.11** Sample size flow chart for physical inactivity and caregiving exit, comparing exit vs non-caregivers and exit vs long-term caregivers. Only participants in the treatment group with complete baseline covariate and health behaviour data were eligible for propensity score matching. Control group participants were drawn from the pool of non-transitioning individuals with complete baseline data on the same variables.

**Figure A6.7**

**Figure S.12** Sample size flow chart for fruit and vegetable consumption and caregiving exit, comparing exit vs non-caregivers and exit vs long-term caregivers. Only participants in the treatment group with complete baseline covariate and health behaviour data were eligible for propensity score matching. Control group participants were drawn from the pool of non-transitioning individuals with complete baseline data on the same variables.

**Figure S.13** Sample size flow chart for problematic drinking and caregiving exit, comparing exit vs non-caregivers and exit vs long-term caregivers. Only participants in the treatment group with complete baseline covariate and health behaviour data were eligible for propensity score matching. Control group participants were drawn from the pool of non-transitioning individuals with complete baseline data on the same variables.

# S5: Descriptive Tables

**Table S.2** Care intensity by age group across outcome-specific analytical samples. Values are n (%) within age group for each outcome-specific caregiver sample at caregiving onset. Differences in totals across outcomes reflect outcome-specific analytical samples.

| **Age group** | **Physical activity** | | | **Diet** | | | **Alcohol** | | | **Smoking** | | |
| --- | --- | --- | --- | --- | --- | --- | --- | --- | --- | --- | --- | --- |
|  | **Total n** | **<20 h n (%)** | **≥20 h n (%)** | **Total n** | **<20 h n (%)** | **≥20 h n (%)** | **Total n** | **<20 h n (%)** | **≥20 h n (%)** | **Total n** | **<20 h n (%)** | **≥20 h n (%)** |
| 16–24 | 269 | 235 (87.4) | 34 (12.6) | 279 | 233 (83.5) | 46 (16.5) | 279 | 233 (83.5) | 46 (16.5) | 954 | 818 (85.7) | 136 (14.3) |
| 25–29 | 177 | 130 (73.5) | 47 (26.6) | 198 | 149 (75.3) | 49 (24.8) | 198 | 149 (75.3) | 49 (24.8) | 426 | 337 (79.1) | 89 (20.9) |
| 30–34 | 239 | 177 (74.1) | 62 (25.9) | 282 | 206 (73.0) | 76 (27.0) | 283 | 207 (73.1) | 76 (26.9) | 516 | 410 (79.5) | 106 (20.5) |
| 35–39 | 332 | 255 (76.8) | 77 (23.2) | 332 | 266 (80.1) | 66 (19.9) | 332 | 266 (80.1) | 66 (19.9) | 605 | 484 (80.0) | 121 (20.0) |
| 40–44 | 380 | 318 (83.7) | 62 (16.3) | 449 | 381 (84.9) | 68 (15.1) | 447 | 379 (84.8) | 68 (15.2) | 861 | 737 (85.6) | 124 (14.4) |
| 45–49 | 541 | 466 (86.1) | 75 (13.9) | 583 | 504 (86.5) | 79 (13.6) | 582 | 503 (86.4) | 79 (13.6) | 1002 | 894 (89.2) | 108 (10.8) |
| 50–54 | 561 | 493 (87.9) | 68 (12.1) | 534 | 475 (89.0) | 59 (11.1) | 535 | 476 (89.0) | 59 (11.0) | 926 | 829 (89.5) | 97 (10.5) |
| 55–59 | 485 | 424 (87.4) | 61 (12.6) | 479 | 403 (84.1) | 76 (15.9) | 480 | 405 (84.4) | 75 (15.6) | 814 | 706 (86.7) | 108 (13.3) |
| 60–64 | 413 | 353 (85.5) | 60 (14.5) | 366 | 318 (86.9) | 48 (13.1) | 367 | 319 (86.9) | 48 (13.1) | 654 | 582 (89.0) | 72 (11.0) |
| 65–69 | 354 | 288 (81.4) | 66 (18.6) | 338 | 268 (79.3) | 70 (20.7) | 338 | 268 (79.3) | 70 (20.7) | 660 | 553 (83.8) | 107 (16.2) |
| 70–74 | 262 | 214 (81.7) | 48 (18.3) | 218 | 170 (78.0) | 48 (22.0) | 217 | 169 (77.9) | 48 (22.1) | 429 | 351 (81.8) | 78 (18.2) |
| 75+ | 250 | 181 (72.4) | 69 (27.6) | 188 | 139 (73.9) | 49 (26.1) | 188 | 139 (73.9) | 49 (26.1) | 469 | 381 (81.2) | 88 (18.8) |
| **Total** | **4,263** | **3,534 (82.9)** | **729 (17.1)** | **4,246** | **3,512 (82.7)** | **734 (17.3)** | **4,246** | **3,513 (82.7)** | **733 (17.3)** | **8,316** | **7,082 (85.2)** | **1,234 (14.8)** |

Table 2

**Table S.3** Caregiving intensity and place of caregiving by life course stage and sex across outcome-specific analytical samples *Values are n (%) unless otherwise stated. Percentages are row percentages within sex, with totals calculated within life course stage.*

| **Outcome** | **Characteristic** | **Early adulthood caregivers (age 16–29 years)** | | | **Early mid-adulthood caregivers (age 30–49 years)** | | | **Late mid-adulthood caregivers (age 50–64 years)** | | | **Later life caregivers (age 65 years and older)** | | |
| --- | --- | --- | --- | --- | --- | --- | --- | --- | --- | --- | --- | --- | --- |
|  |  | **Total** | **Men** | **Women** | **Total** | **Men** | **Women** | **Total** | **Men** | **Women** | **Total** | **Men** | **Women** |
| **Physical activity** | **Became caregiver** | **446** | **157** | **289** | **1492** | **542** | **950** | **1459** | **614** | **845** | **866** | **409** | **457** |
|  | Low intensity (≤20 hrs/week) | 365 (81.8%) | 139 (88.5%) | 226 (78.2%) | 1,216 (81.5%) | 459 (84.7%) | 757 (79.7%) | 1,270 (87.1%) | 545 (88.8%) | 725 (85.8%) | 683 (78.9%) | 320 (78.2%) | 363 (79.4%) |
|  | High intensity (>20 hours/week) | 81 (18.2%) | 18 (11.5%) | 63 (21.8%) | 276 (18.5%) | 83 (15.3%) | 193 (20.3%) | 189 (13.0%) | 69 (11.2%) | 120 (14.2%) | 183 (21.1%) | 89 (21.8%) | 94 (20.6%) |
|  | Outside household | 234 (50.9%) | 70 (42.4%) | 164 (55.6%) | 1,082 (70.1%) | 355 (62.6%) | 727 (74.4%) | 1,124 (74.7%) | 441 (69.9%) | 683 (78.2%) | 463 (50.0%) | 209 (47.8%) | 254 (51.9%) |
|  | Inside household | 190 (41.3%) | 73 (44.2%) | 117 (39.7%) | 398 (25.8%) | 186 (32.8%) | 212 (21.7%) | 316 (21.0%) | 165 (26.2%) | 151 (17.3%) | 421 (45.5%) | 209 (47.8%) | 212 (43.4%) |
|  | Inside and outside household | 36 (7.8%) | 22 (13.3%) | 14 (4.8%) | 64 (4.2%) | 26 (4.6%) | 38 (3.9%) | 64 (4.3%) | 25 (4.0%) | 39 (4.5%) | 42 (4.5%) | 19 (4.4%) | 23 (4.7%) |
| **Diet** | **Became caregiver** | **452** | **158** | **294** | **1498** | **546** | **952** | **1469** | **619** | **850** | **876** | **411** | **465** |
|  | Low intensity (≤20 hrs/week) | 371 (82.1%) | 140 (88.6%) | 231 (78.6%) | 1,221 (81.5%) | 463 (84.8%) | 758 (79.6%) | 1,277 (86.9%) | 550 (88.9%) | 727 (85.5%) | 690 (78.8%) | 322 (78.3%) | 368 (79.1%) |
|  | High intensity (>20 hours/week) | 81 (17.9%) | 18 (11.4%) | 63 (21.4%) | 277 (18.5%) | 83 (15.2%) | 194 (20.4%) | 192 (13.1%) | 69 (11.2%) | 123 (14.5%) | 186 (21.2%) | 89 (21.7%) | 97 (20.9%) |
|  | Outside household | 238 (51.1%) | 71 (42.8%) | 167 (55.7%) | 1,086 (70.1%) | 357 (62.5%) | 729 (74.5%) | 1,132 (74.8%) | 444 (69.8%) | 688 (78.4%) | 468 (50.0%) | 211 (48.1%) | 257 (51.7%) |
|  | Inside household | 191 (41.0%) | 73 (44.0%) | 118 (39.3%) | 399 (25.7%) | 187 (32.8%) | 212 (21.7%) | 318 (21.0%) | 166 (26.1%) | 152 (17.3%) | 425 (45.4%) | 209 (47.6%) | 216 (43.5%) |
|  | Inside and outside household | 37 (7.9%) | 22 (13.3%) | 15 (5.0%) | 65 (4.2%) | 27 (4.7%) | 38 (3.9%) | 64 (4.2%) | 26 (4.1%) | 38 (4.3%) | 43 (4.6%) | 19 (4.3%) | 24 (4.8%) |
| **Alcohol** | **Became caregiver** | **452** | **158** | **294** | **1496** | **544** | **952** | **1472** | **619** | **853** | **875** | **411** | **464** |
|  | Low intensity (≤20 hrs/week) | 371 (82.1%) | 140 (88.6%) | 231 (78.6%) | 1,219 (81.5%) | 461 (84.7%) | 758 (79.6%) | 1,281 (87.0%) | 551 (89.0%) | 730 (85.6%) | 689 (78.7%) | 322 (78.4%) | 367 (79.1%) |
|  | High intensity (>20 hours/week) | 81 (17.9%) | 18 (11.4%) | 63 (21.4%) | 277 (18.5%) | 83 (15.3%) | 194 (20.4%) | 191 (13.0%) | 68 (11.0%) | 123 (14.4%) | 186 (21.3%) | 89 (21.7%) | 97 (20.9%) |
|  | Outside household | 238 (51.1%) | 71 (42.8%) | 167 (55.7%) | 1,083 (70.0%) | 355 (62.4%) | 728 (74.4%) | 1,134 (74.8%) | 444 (69.8%) | 690 (78.3%) | 466 (49.8%) | 210 (47.8%) | 256 (51.6%) |
|  | Inside household | 191 (41.0%) | 73 (44.0%) | 118 (39.3%) | 400 (25.8%) | 187 (32.9%) | 213 (21.8%) | 318 (21.0%) | 166 (26.1%) | 152 (17.3%) | 426 (45.6%) | 210 (47.8%) | 216 (43.5%) |
|  | Inside and outside household | 37 (7.9%) | 22 (13.3%) | 15 (5.0%) | 65 (4.2%) | 27 (4.8%) | 38 (3.9%) | 65 (4.3%) | 26 (4.1%) | 39 (4.4%) | 43 (4.6%) | 19 (4.3%) | 24 (4.8%) |
| **Smoking** | **Became caregiver** | **1373** | **548** | **825** | **2978** | **1120** | **1858** | **2404** | **1057** | **1347** | **1561** | **710** | **851** |
|  | Low intensity (≤20 hrs/week) | 1,151 (83.8%) | 495 (90.3%) | 656 (79.5%) | 2,517 (84.5%) | 986 (88.0%) | 1,531 (82.4%) | 2,127 (88.5%) | 948 (89.7%) | 1,179 (87.5%) | 1,287 (82.5%) | 580 (81.7%) | 707 (83.1%) |
|  | High intensity (>20 hours/week) | 222 (16.2%) | 53 (9.7%) | 169 (20.5%) | 461 (15.5%) | 134 (12.0%) | 327 (17.6%) | 277 (11.5%) | 109 (10.3%) | 168 (12.5%) | 274 (17.6%) | 130 (18.3%) | 144 (16.9%) |
|  | Outside household | 798 (55.4%) | 288 (49.5%) | 510 (59.4%) | 2,287 (74.2%) | 821 (70.1%) | 1,466 (76.7%) | 1,905 (76.3%) | 803 (72.9%) | 1,102 (79.1%) | 943 (57.6%) | 420 (56.4%) | 523 (58.6%) |
|  | Inside household | 573 (39.8%) | 268 (46.0%) | 305 (35.5%) | 700 (22.7%) | 307 (26.2%) | 393 (20.6%) | 521 (20.9%) | 273 (24.8%) | 248 (17.8%) | 656 (40.0%) | 306 (41.1%) | 350 (39.2%) |
|  | Inside and outside household | 69 (4.8%) | 26 (4.5%) | 43 (5.0%) | 96 (3.1%) | 44 (3.8%) | 52 (2.7%) | 70 (2.8%) | 26 (2.4%) | 44 (3.2%) | 39 (2.4%) | 19 (2.6%) | 20 (2.2%) |

# S6: Descriptive Outcome by Wave

**Table S.4** Cross-sectional prevalence of health behaviours by UKHLS wave, caregiving status, and caregiving hours. Values represent the prevalence (%) of physical inactivity, problematic drinking, and smoking, and the mean daily portions of fruit and vegetable consumption, among participants who reported caregiving status and the respective health behaviour at least once during the study period. Data from the UK Household Longitudinal Study (UKHLS). Physical inactivity, diet, and problematic drinking were measured at waves 7, 9, 11, and 13. Smoking was measured at waves 5 through 13. N indicates the number of participants with valid data at each wave. Caregiving hours refer to weekly hours of unpaid care provided. Values are unweighted cross-sectional estimates.

|  |  |  |  | **Caregiving hours** | |
| --- | --- | --- | --- | --- | --- |
| **UKHLS wave** | **N** | **Caregiver** | **Non-caregiver** | **<20 hrs/week** | **≥20 hrs/week** |
| **Physical inactivity (% inactive) (N = 47,524)** | | | | | |
| 7 | 42,120 | 55.7 | 56.4 | 53.9 | 63.3 |
| 9 | 36,025 | 51.2 | 49.8 | 46.0 | 59.6 |
| 11 | 30,543 | 53.3 | 52.8 | 50.1 | 59.4 |
| 13 | 29,907 | 54.2 | 54.0 | 50.8 | 61.2 |
| **Fruit and vegetable consumption (mean daily portions) (N = 47,666)** | | | | | |
| 7 | 39,170 | 3.6 | 3.4 | 3.7 | 3.3 |
| 9 | 34,375 | 3.8 | 3.6 | 3.8 | 3.5 |
| 11 | 29,700 | 3.7 | 3.6 | 3.9 | 3.3 |
| 13 | 27,017 | 3.6 | 3.5 | 3.8 | 3.3 |
| **Problematic drinking (% problematic) (N = 46,929)** | | | | | |
| 7 | 39,600 | 46.6 | 49.1 | 50.2 | 36.6 |
| 9 | 35,096 | 48.0 | 49.8 | 51.4 | 39.1 |
| 11 | 30,524 | 43.8 | 47.7 | 47.7 | 34.2 |
| 13 | 27,809 | 42.6 | 45.0 | 47.0 | 33.0 |
| **Smoking (% current smoker) (N = 57,498)** | | | | | |
| 5 | 42,729 | 19.7 | 17.6 | 17.7 | 25.3 |
| 6 | 39,203 | 19.7 | 16.1 | 17.8 | 25.2 |
| 7 | 40,852 | 17.3 | 15.2 | 15.4 | 22.7 |
| 8 | 35,509 | 16.3 | 14.2 | 14.5 | 20.5 |
| 9 | 35,570 | 15.4 | 13.0 | 13.6 | 20.7 |
| 10 | 33,938 | 14.3 | 12.8 | 13.0 | 17.8 |
| 11 | 30,542 | 13.4 | 11.9 | 11.8 | 17.4 |
| 12 | 29,115 | 12.2 | 10.8 | 11.2 | 14.8 |
| 13 | 27,864 | 11.8 | 10.2 | 9.6 | 17.4 |

# S7: Distribution of caregiving transitions by outcomes

**Table S.5** Distribution of caregiving transitions by UKHLS wave, separately for caregiving entry and exit and by health behaviour outcome. The table shows the number and proportion of matched participants (treated group) who first transitioned into or out of unpaid caregiving at each wave of the UK Household Longitudinal Study. Transition period indicates the interval between consecutive UKHLS waves during which the participant first reported a change in caregiving status. For caregiving entry, this is the first wave at which a previously non-caregiving participant reported providing unpaid care. For caregiving exit, this is the first wave at which a previously caregiving participant reported no longer providing care. Smoking was measured at every wave (UKHLS waves 5–13, nine waves), while physical inactivity, diet, and problematic drinking were measured biennially (waves 7, 9, 11, 13, four waves). Approximate fieldwork years are based on the UKHLS data collection schedule. Samples are restricted to matched treated participants from the propensity score matched analyses for each outcome.

|  |  | **Caregiving entry** | | **Caregiving exit** | |
| --- | --- | --- | --- | --- | --- |
| **Transition period** | **Approx. fieldwork** | **N** | **%** | **N** | **%** |
| **Smoking** | | | | | |
| Wave 5 → 6 | 2013–2015 | 2,532 | 29.2 | 2,028 | 37.7 |
| Wave 6 → 7 | 2014–2016 | 1,202 | 13.9 | 1,180 | 21.9 |
| Wave 7 → 8 | 2015–2017 | 1,169 | 13.5 | 682 | 12.7 |
| Wave 8 → 9 | 2016–2018 | 1,005 | 11.6 | 479 | 8.9 |
| Wave 9 → 10 | 2017–2019 | 817 | 9.4 | 363 | 6.7 |
| Wave 10 → 11 | 2018–2020 | 677 | 7.8 | 269 | 5.0 |
| Wave 11 → 12 | 2019–2021 | 694 | 8.0 | 209 | 3.9 |
| Wave 12 → 13 | 2020–2022 | 563 | 6.5 | 175 | 3.2 |
| **Total** |  | **8,659** | **100.0** | **5,385** | **100.0** |
| **Physical inactivity** | | | | | |
| Wave 7 → 9 | 2015–2019 | 2,166 | 48.8 | 1,975 | 59.1 |
| Wave 9 → 11 | 2017–2021 | 1,257 | 28.3 | 843 | 25.2 |
| Wave 11 → 13 | 2019–2023 | 1,013 | 22.8 | 522 | 15.6 |
| **Total** |  | **4,436** | **100.0** | **3,340** | **100.0** |
| **Diet (fruit and vegetable consumption)** | | | | | |
| Wave 7 → 9 | 2015–2019 | 2,181 | 48.8 | 1,990 | 59.2 |
| Wave 9 → 11 | 2017–2021 | 1,265 | 28.3 | 850 | 25.3 |
| Wave 11 → 13 | 2019–2023 | 1,022 | 22.9 | 523 | 15.5 |
| **Total** |  | **4,468** | **100.0** | **3,363** | **100.0** |
| **Problematic drinking** | | | | | |
| Wave 7 → 9 | 2015–2019 | 2,178 | 48.7 | 1,994 | 59.2 |
| Wave 9 → 11 | 2017–2021 | 1,269 | 28.4 | 851 | 25.2 |
| Wave 11 → 13 | 2019–2023 | 1,021 | 22.8 | 526 | 15.6 |
| **Total** |  | **4,468** | **100.0** | **3,371** | **100.0** |

# S8: Covariate Balance

**Table S.6** Covariate balance before matching, after propensity score matching (PSM), and after entropy balancing for smoking and transition into caregiving. The pre-matching sample includes all participants with at least two waves of observation on caregiving status and the outcome variable, and complete data on baseline covariates. Values are percentages for categorical variables and means for continuous variables. p-values are from chi-squared tests (categorical) or t-tests (continuous) for the before-matching comparison, and from weighted regression F-tests for PSM and entropy-balanced comparisons. Control = non-caregivers; Treated = transitioned into caregiving. PSM = propensity score matching (1:3 nearest neighbour, exact matching on sex, age, and baseline wave). Entropy balancing was applied to the PSM sample to correct for residual covariate imbalance.

|  |  | **Before matching** | | | **After PSM** | | | **After entropy balancing** | | |
| --- | --- | --- | --- | --- | --- | --- | --- | --- | --- | --- |
| **Variable** | **Category** | **Control** | **Treated** | **p** | **Control** | **Treated** | **p** | **Control** | **Treated** | **p** |
| **Age at baseline** | Mean | 41.55 | 48.00 | <0.001 | 46.97 | 46.90 | 0.764 | 48.06 | 48.06 | 0.999 |
| **GHQ-12 score** | Mean | 10.78 | 11.28 | <0.001 | 10.96 | 11.17 | 0.013 | 11.28 | 11.28 | 1.000 |
| **Number of waves** | Mean | 6.0 | 7.35 | <0.001 | 7.17 | 7.16 | 0.871 | 7.35 | 7.35 | 0.999 |
| **Sex** | Male | 47.5 | 41.6 | 0.004 | 43.2 | 43.2 | 0.995 | 41.6 | 41.6 | 1.000 |
|  | Female | 52.5 | 58.4 |  | 56.8 | 56.8 |  | 58.4 | 58.4 |  |
| **Cohabiting status** | Single, separated, widowed | 46.0 | 32.9 | <0.001 | 35.7 | 35.1 | 0.344 | 32.9 | 32.9 | 1.000 |
|  | Married or cohabiting | 54.0 | 67.1 |  | 64.3 | 64.9 |  | 67.1 | 67.1 |  |
| **Household size** | 1 | 14.2 | 12.5 | <0.001 | 15.3 | 12.7 | <0.001 | 12.5 | 12.5 | 1.000 |
|  | 2 | 28.6 | 35.9 |  | 33.7 | 34.2 |  | 35.9 | 35.9 |  |
|  | 3–4 | 40.9 | 38.4 |  | 38.6 | 39.0 |  | 38.4 | 38.4 |  |
|  | 5 or more | 16.4 | 13.2 |  | 12.5 | 14.1 |  | 13.2 | 13.2 |  |
| **Highest qualification** | No qualification | 11.3 | 10.3 | <0.001 | 10.8 | 10.3 | 0.003 | 10.3 | 10.3 | 1.000 |
|  | A-Level, GCSE, other qual. | 53.7 | 52.3 |  | 50.4 | 52.8 |  | 52.3 | 52.3 |  |
|  | Degree or other higher | 35.0 | 37.3 |  | 38.8 | 36.9 |  | 37.3 | 37.3 |  |
| **Occupational class** | Not employed | 46.4 | 42.2 | <0.001 | 41.8 | 42.8 | 0.010 | 42.2 | 42.2 | 1.000 |
|  | Management & professional | 23.9 | 25.0 |  | 26.6 | 24.7 |  | 25.0 | 25.0 |  |
|  | Intermediate | 12.2 | 13.8 |  | 13.9 | 13.6 |  | 13.8 | 13.8 |  |
|  | Routine | 17.5 | 19.0 | <0.001 | 17.8 | 18.8 | 0.03 | 19.0 | 19.0 | 0.637 |
| **Employment status** | Unemployed | 40.8 | 39.1 |  | 37.9 | 39.6 |  | 38.7 | 39.1 |  |
|  | Full-time | 43.7 | 43.6 |  | 45.5 | 43.8 |  | 44.2 | 43.6 | 1.000 |
|  | Part-time | 15.5 | 17.3 |  | 16.6 | 16.6 |  | 17.0 | 17.3 |  |
| **Income quintile** | 1 (lowest) | 18.3 | 18.5 | 0.02 | 17.4 | 18.8 | <0.001 | 19.0 | 18.5 | 0.770 |
|  | 2 | 19.5 | 20.9 |  | 19.4 | 21.0 |  | 20.3 | 20.9 |  |
|  | 3 | 20.2 | 20.2 |  | 20.2 | 20.2 |  | 20.2 | 20.2 |  |
|  | 4 | 20.8 | 20.2 |  | 21.0 | 20.1 |  | 20.3 | 20.2 |  |
|  | 5 (highest) | 21.3 | 20.1 |  | 22.0 | 19.9 |  | 20.2 | 20.1 |  |
| **Ethnicity** | White | 81.2 | 85.1 | <0.001 | 85.0 | 84.1 | 0.004 | 85.1 | 85.1 | 1.000 |
|  | Black | 5.9 | 4.6 |  | 4.7 | 4.8 |  | 4.6 | 4.6 |  |
|  | Indian | 3.9 | 3.1 |  | 3.1 | 3.4 |  | 3.1 | 3.1 |  |
|  | Pakistani/Bangladeshi | 5.1 | 4.6 |  | 4.0 | 5.0 |  | 4.6 | 4.6 |  |
|  | Other Asian/other | 3.9 | 2.6 |  | 3.2 | 2.8 |  | 2.6 | 2.6 |  |
| **Self-rated health** | Excellent/very good/good | 83.8 | 80.5 | <0.001 | 81.7 | 81.4 | 0.562 | 80.5 | 80.5 | 1.000 |
|  | Fair/poor | 16.2 | 19.5 |  | 18.3 | 18.6 |  | 19.5 | 19.5 |  |
| **Smoker at baseline** | No | 84.0 | 82.8 | 0.009 | 83.9 | 82.4 | 0.006 | 82.8 | 82.8 | 1.000 |
|  | Yes | 16.0 | 17.2 |  | 16.1 | 17.6 |  | 17.2 | 17.2 |  |
| **N** |  | 25,915 | 8,655 |  | **17,316** | **8,659** |  | **17,314** | **8,658** |  |

# S9: Coefficients and p-values for piecewise regression

**Table S.7: P-values for slope differences in health behaviour trajectories from piecewise growth curve models for transition into caregiving. Represents** p-values for slope differences between participants who transitioned into unpaid caregiving and propensity score matched non-caregivers, estimated from piecewise growth curve models. Slope comparisons are made for two distinct time segments: the transition period (from one or two years before to the caregiving onset year, “-1 to 0” or “–2 to 0”) and the post-transition period (from caregiving onset to four years after for physical activity, diet, and alcohol; and up to seven years for smoking). Outcomes include physical activity, fruit and vegetable consumption (diet), problematic drinking, and smoking. Models are stratified by caregiving intensity, place of care, sex, and age group. All estimates are derived from models fitted to matched samples and account for baseline differences through propensity score matching.

| **Physical activity** | **(n entering caregiving)** |  | **Global p-values (wald test)** |  | **Post-transition period**  **(“0 to 4”** |
| --- | --- | --- | --- | --- | --- |
| Caregiving status | 4,436 | -0.09 (-0.17/-0.02) | 0.02 | 0.12 (0.06/0.18) | 0.04 |
| Caregiving intensity | 4,263 |  |  |  |  |
| Low <20 hours |  | -0.09 (-0.17/-0.01) | 0.05 | 0.12 (0.00/0.24) |  |
| High > 20 hours |  | -0.10 (-0.27/0.07) |  | 0.18 (-0.08/0.43) | 0.07 |
| Matched low & high intensity |  | 0.17 (-0.02/0.36) | 0.08 | -0.14 (-0.42/0.15) | 0.35 |
| Place of caregiving | 4,434 |  |  |  |  |
| Outside household |  | -0.15 (-0.23/-0.08) |  | 0.14 (0.01/0.27) |  |
| Inside household |  | -0.06 (-0.19/0.06) |  | 0.15 (-0.04/0.33) |  |
| Dual (outside and inside household) |  | 0.05 (-0.30/0.39) | 0.05 | 0.11 (-0.40/0.62) | 0.11 |
| **Transition*sex** | **4,436** |  | **0.83** |  | **0.88** |
| Male only | 1,800 | -0.10 (-0.22/0.01) | 0.08 | 0.16 (-0.02/0.33) | 0.08 |
| Female only | 2,636 | -0.09 (-0.18/0.01) | 0.07 | 0.09 (-0.05/0.23) | 0.21 |
| **Transition*age group** | **4,436** |  | **0.95** |  | **0.29** |
| Early adulthood (16-29) | 461 | -0.07 (-0.31/0.17) | 0.77 | 0.04 (-0.32/0.41) | 0.94 |
| Early mid-adulthood (30-49) | 1,544 | -0.07 (-0.20/0.06) | 0.28 | 0.17 (-0.02/0.36) | 0.08 |
| Late mid-adulthood (50-64) | 1,505 | -0.07 (-0.20/0.06) | 0.23 | -0.02 (-0.20/0.17) | 0.97 |
| Late adulthood (65+) | 926 | -0.20 (-0.36/-0.04) | 0.02 | 0.28 (0.05/0.52) | 0.02 |
| **Diet** | **(n entering caregiving)** |  | **Transition period**  **(“-2 to 0”)** |  | **Post-transition period**  **(“0 to 4”)** |
| Caregiving status | 4,692 | 0.02 (-0.05/0.09) | 0.58 | -0.01 (-0.12/0.09) | 0.80 |
| Caregiving intensity | 4,295 |  |  |  |  |
| Low |  | 0.09 (0.01/0.18) |  | 0.00 (-0.11/0.11) |  |
| high |  | 0.02 (-0.13/0.18) | 0.93 | -0.09 (-0.33/0.14) | 0.73 |
| Matched low & high intensity |  | -0.04 (-0.21/0.14) | 0.69 | 0.00 (-0.26/0.26) | 0.99 |
| Place of caregiving | 4,466 |  |  |  |  |
| Outside household |  | 0.02 (-0.06/0.11) |  | -0.01 (-0.13/0.10) |  |
| Inside household |  | 0.03 (-0.09/0.14) |  | -0.06 (-0.23/0.12) |  |
| Dual (inside and outside household) |  | -0.04 (-0.55/0.08) | 0.88 | -0.07 (-0.44/0.31) | 0.90 |
| **Transition*sex** | **4,692** |  | **0.17** |  | **0.13** |
| Male only | 1,812 | -0.01 (-0.12/0.10) | 0.97 | 0.01 (-0.16/0.17) | 0.97 |
| Female only | 2,656 | 0.00 (-0.11/0.10) | 0.89 | -0.01 (-0.17/0.15) | 0.86 |
| **Transition*age group** | **4,692** |  | **0.06** |  | **0.73** |
| Early adulthood (16-29) | 467 | -0.09 (-0.29/0.11) | 0.28 | 0.02 (-0.28/0.32) | 0.75 |
| Early mid-adulthood (30-49) | 1,550 | -0.13 (-0.25/-0.01) | 0.03 | 0.15 (-0.02/0.33) | 0.09 |
| Late mid-adulthood (50-64) | 1,515 | 0.11 (-0.01/0.24) | 0.06 | -0.08 (-0.25/0.10) | 0.39 |
| Late adulthood (65+) | 936 | 0.09 (-0.06/0.23) | 0.24 | -0.05 (-0.26/0.16) | 0.64 |
| **Problematic drinking** | **(n entering caregiving)** |  | **Transition period**  **(“-2 to 0”)** |  | **Post-transition period**  **(“0 to 4”)** |
| Caregiving status | 4,468 | 0.01 (-0.05/0.07) | 0.77 | -0.04 (-0.12/0.04) | 0.35 |
| Caregiving intensity | 4,295 |  |  |  |  |
| Low |  | 0.02 (-0.04/0.09) |  | -0.05 (-0.14/0.04) |  |
| high |  | -0.04 (-0.17/0.09) | 0.61 | -0.08 (-0.28/0.12) | 0.46 |
| Matched low & high intensity |  | -0.09 (-0.23/0.05) | 0.21 | -0.01 (-0.22/0.21) | 0.95 |
| Place of caregiving | 4,295 |  |  |  |  |
| Outside |  | 0.02 (-0.05/0.09) |  | -0.07 (-0.16/0.03) |  |
| Inside |  | -0.03 (-0.12/0.07) |  | 0.01 (-0.13/0.16) |  |
| Dual |  | 0.12 (-0.11/0.35) | 0.60 | -0.20 (-0.55/0.15) | 0.37 |
| **Transition*sex** | **4,468** |  | **0.37** |  | **0.27** |
| Male only | 1,810 | 0.02 (-0.08/0.11) | 0.56 | -0.01 (-0.15/0.13) | 0.75 |
| Female only | 2,658 | -0.05 (-0.13/0.03) | 0.20 | 0.06 (-0.06/0.17) | 0.31 |
| **Transition*age group** | **4,468** |  | **0.78** |  | **0.33** |
| Early adulthood (16-29) | 467 | -0.11 (-0.31/0.15) | 0.34 | 0.21 (-0.14/0.57) | 0.24 |
| Early mid-adulthood (30-49) | 1,548 | -0.02 (-0.13/0.09) | 0.71 | -0.08 (-0.24/0.08) | 0.32 |
| Late mid-adulthood (50-64) | 1,518 | 0.06 (-0.08/0.10) | 0.27 | -0.07 (-0.21/0.07) | 0.31 |
| Late adulthood (65+) | 935 | -0.02 (-0.16/0.11) | 0.60 | 0.07 (-0.12/0.26) | 0.52 |
| **Smoking** | **(n entering caregiving)** |  | **Transition period**  **(“-1 to 0”)** |  | **Post-transition period**  **(“0 to 7”)** |
| Caregiving status | 8,659 | 0.13 (0.07/0.20) | <0.001 | -0.13 (-0.20/-0.06) | <0.001 |
| Caregiving intensity | 8,316 |  |  |  |  |
| Low |  | 0.11 (0.04/0.18) |  | -0.10 (-0.17/0.03) |  |
| high |  | 0.21 (0.09/0.32) | <0.001 | -0.21 (-0.34/-0.09) | <0.001 |
| Matched low & high intensity |  | 0.12 (-0.01/0.25) | 0.08 | -0.13 (-0.27/0.01) | 0.07 |
| Place of caregiving | 8,657 |  |  |  |  |
| Outside |  | 0.09 (0.02/0.17) |  | -0.08 (-0.16/0.00) |  |
| Inside |  | 0.21 (0.11/0.31) |  | -0.21 (-0.31/-0.10) |  |
| Dual |  | 0.19 (-0.10/0.48) | <0.001 | -0.20 (-0.50/0.10) | 0.001 |
| **Transition*sex** | **8,659** |  | **0.82** |  | **0.81** |
| Male only | 3,601 | 0.05 (-0.05/0.15) | 0.32 | -0.04 (-0.15/0.07) | 0.45 |
| Female only | 5,058 | 0.04 (-0.06/0.14) | 0.11 | -0.03 (-0.14/0.08) | 0.26 |
| **Transition*age group** | **8,659** |  | **0.02** |  | **0.05** |
| Early adulthood (16-29) | 1,441 | 0.17 (0.02/0.33) | 0.03 | -0.17 (-0.36/0.01) | 0.06 |
| Early mid-adulthood (30-49) | 3,083 | 0.20 (0.09/0.30) | <0.001 | -0.16 (-0.28/-0.04) | 0.01 |
| Late mid-adulthood (50-64) | 2,497 | -0.04 (-0.17/0.09) | 0.43 | 0.05 (-0.09/0.19) | 0.29 |
| Late adulthood (65+) | 1,638 | 0.04 (-0.17/0.25) | 0.21 | 0.03 (-0.20/0.27) | 0.75 |

**Table S.8: P-values for slope differences in health behaviour trajectories following cessation of unpaid caregiving: comparisons with continuing caregivers and non-caregivers. Represents** p-values for slope differences in health behaviour trajectories estimated from piecewise growth curve models, comparing individuals who exited unpaid caregiving with two propensity score-matched control groups: (1) those who continued caregiving, and (2) non-caregivers. Slope comparisons are made for two distinct time segments: the transition period (from one or two years before to the caregiving cessation year, “-1 to 0” or “–2 to 0”) and the post-transition period (from caregiving onset to four years after for physical activity, diet, and alcohol; and up to seven years for smoking). Outcomes include physical activity, fruit and vegetable consumption (diet), problematic drinking, and smoking. Models are stratified by caregiving intensity, place of care, sex, and age group. All estimates are derived from models fitted to matched samples and account for baseline differences through propensity score matching.

|  |  |  |  | | Exit caregiving vs. continuing caregiving | |
| --- | --- | --- | --- | --- | --- | --- |
| **Physical inactivity** | **(n exit)** |  | **Transition period**  **(“-2 to 0”)** |  | | **Post-transition period**  **(“2 to 4”)** |
| Caregiving status | 3,340 | 0.12 (-0.01/0.28) | 0.07 | -0.18 (-0.40/0.03) | | 0.10 |
| Caregiving intensity | 3,307 |  |  |  | |  |
| low |  | -0.03 (-0.16/0.09) |  | -0.09 (-0.32/0.13) | |  |
| high |  | 0.36 (0.17/0.54) | 0.01 | -0.49 (-0.82/-0.16) | | 0.01 |
| Place of caregiving | 3,330 |  |  |  | |  |
| Outside |  | 0.10 (-0.06/0.25) |  | -0.09 (-0.32/0.14) | |  |
| Inside |  | 0.23 (0.03/0.44) |  | -0.38 (-0.67/-0.08) | |  |
| Dual |  | 0.14 (-0.22/0.51) | 0.16 | -0.20 (-0.76/0.36) | | 0.09 |
| **Transition*sex** | **3,340** |  | **0.75** |  | | **0.92** |
| Male only | 1,247 | 0.39 (0.14/0.63) | 0.002 | -0.61 (-0.98/-0.25) | | 0.001 |
| Female only | 2,093 | 0.29 (0.10/0.49) | 0.003 | -0.36 (-0.64/-0.08) | | 0.02 |
| **Transition*age group** | **3,340** |  | **0.45** |  | | **0.82** |
| Early adulthood (16-29) | 287 | -0.28 (-0.97/0.40) | 0.42 | -0.12 (-1.29/1.04) | | 0.84 |
| Early mid-adulthood (30-49) | 874 | 0.29 (-0.02/0.59) | 0.06 | -0.37 (-0.80/0.06) | | 0.09 |
| Late mid-adulthood (50-64) | 1,191 | -0.04 (-0.26/0.19) | 0.76 | 0.21 (-0.12/0.54) | | 0.22 |
| Late adulthood (65+) | 988 | 0.26 (-0.10/0.63) | 0.16 | -0.42 (-0.15/0.10) | | 0.11 |
| **Diet** | **(n exit)** |  | **Transition period**  **(“-2 to 0”)** |  | | **Post-transition period**  **(“2 to 4”)** |
| Caregiving status | 3,363 | -0.05 (-0.18/0.08) | 0.50 | 0.09 (-0.09/0.28) | | 0.33 |
| Caregiving intensity | 3,331 |  |  |  | |  |
| low |  | -0.04 (-0.18/0.09) |  | 0.06 (-0.12/0.25) | |  |
| high |  | -0.07 (-0.26/0.12) | 0.71 | 0.20 (-0.0.08/0.49) | | 0.39 |
| Place of caregiving | 3,353 |  |  |  | |  |
| Outside |  | -0.05 (-0.19/0.09) |  | 0.04 (-0.16/0.230 | |  |
| Inside |  | -0.05 (-0.22/0.12) |  | 0.25 (0.00/0.50) | |  |
| Dual |  | 0.00 (-0.34/0.35) | 0.89 | -0.11 (-0.67/0.44) | | 0.18 |
| **Transition*sex** | **3,363** |  | **0.75** |  | | **0.92** |
| Male only | 1,256 | -0.01 (-0.21/0.20) | 0.95 | 0.03 (-0.26/0.32) | | 0.82 |
| Female only | 2,107 | -0.21 (-0.37/-0.04) | 0.02 | 0.29 (0.05/0.53) | | 0.02 |
| **Transition*age group** | **3,363** |  | **0.58** |  | | **0.45** |
| Early adulthood (16-29) | 287 | 0.20 (-0.51/0.92) | 0.58 | -0.02 (-0.97/0.93) | | 0.97 |
| Early mid-adulthood (30-49) | 879 | 0.06 (-0.18/0.29) | 0.64 | -0.05 (-0.40/0.30) | | 0.77 |
| Late mid-adulthood (50-64) | 1,197 | -0/22 (-0.42/-0.01) | 0.04 | 0.27 (-0.02/0/56) | | 0.07 |
| Late adulthood (65+) | 1,000 | 0.10 (-0.17/0.36) | 0.56 | 0.18 (-0.21/0.56) | | 0.37 |
| **Problematic drinking** | **(n exit)** |  | **Transition period**  **(“-2 to 0”)** |  | | **Post-transition period**  **(“2 to 4”)** |
| Caregiving status | 3,371 | 0.12 (0.01/0.23) | 0.04 | -0.15 (-0.31/0.00) | | 0.05 |
| Caregiving intensity | 3,337 |  |  |  | | 0.12 |
| Low |  | 0.10 (-0.01/0.21) |  | -0.16 (-0.32/0.00) | |  |
| high |  | 0.11 (-0.06/0.29) | 0.19 | -0.15 (-0.40/0.10) | | 0.14 |
| Place of caregiving | 3,361 |  |  |  | |  |
| Outside |  | 0.13 (0.02/0.25) |  | -0.19 (-0.35/-0.03) | |  |
| Inside |  | 0.05 (-0.10/0.21) |  | -0.09 (-0.31/0.12) | |  |
| dual |  | 0.03 (-0.26/0.32) | 0.15 | -0.08 (-0.50/0.34) | | 0.12 |
| **Transition*sex** | **3,371** |  | **0.38** |  | | **0.19** |
| Male only | 1,259 | 0.02 (-0.16/0.20) | 0.84 | -0.07 (-0.33/0.18) | | 0.56 |
| Female only | 2,112 | 0.05 (-0.08/0.19) | 0.44 | -0.03 (-0.22/0.16) | | 0.73 |
| **Transition*age group** | **3,371** |  | **0.42** |  | | **0.87** |
| Early adulthood (16-29) | 288 | 0.16 (-0.38/0.70) | 0.57 | -0.07 (-0.91/0.77) | | 0.87 |
| Early mid-adulthood (30-49) | 882 | 0.18 (-0.03/0.39) | 0.09 | -0.13 (-0.40/0.15) | | 0.37 |
| Late mid-adulthood (50-64) | 1,199 | 0.10 (-0.08/0.27) | 0.27 | -0.17 (-0.41/0.06) | | 0.15 |
| Late adulthood (65+) | 1,002 | -0.06 (-0.30/0.18) | 0.62 | 0.00 (-0.30/0.31) | | 0.98 |
| **Smoking** | **(n exit)** |  | **Transition period**  **(“-1 to 0”)** |  | | **Post-transition period**  **(“1 to 7”)** |
| Caregiving status | 5,385 | -0.03 (-0.17/0.12) | 0.72 | 0.01 (-0.14/0.16) | | 0.88 |
| Caregiving intensity | 5,338 |  |  |  | |  |
| low |  | 0.00 (-0.15/0.14) |  | -0.01 (0.16/0.14) | |  |
| high |  | -0.06 (-0.25/0.13) | 0.76 | 0.06 (-0.14/0.27) | | 0.66 |
| Place of caregiving | 5,370 |  |  |  | |  |
| Outside |  | -0.01 (-0.16/0.14) |  | -0.01 (-0.16/0.15) | |  |
| Inside |  | -0.06 (-0.24/0.11) |  | 0.07 (-0.11/0.25) | |  |
| Dual |  | 0.03 (-0.29/0.35) | 0.84 | -0.07 (-0.42/0.27) | | 0.68 |
| **Transition*sex** | **5,385** |  | **0.73** |  | | **0.53** |
| Male only | 2,146 | -0.08 #9-0.33/0.16) | 0.51 | 0.09 (-0.17/0.36) | | 0.49 |
| Female only | 3,239 | -0.23 (-0.43/-0.03) | 0.03 | 0.19 (-0.01/0.39) | | 0.06 |
| **Transition*age group** | **5,385** |  | **0.23** |  | | **0.31** |
| Early adulthood (16-29) | 866 | -0.33 (-0.73/0.07) | 0.11 | 0.33 (-0.15/0.81) | | 0.18 |
| Early mid-adulthood (30-49) | 1,570 | -0.20 (-0.42/0.03) | 0.08 | 0.15 (-0.07/0.37) | | 0.19 |
| Late mid-adulthood (50-64) | 1,776 | -0.05 (-0.33/0.23) | 0.73 | 0.08 (-0.21/0.36) | | 0.59 |
| Late adulthood (65+) | 1,173 | 0.04 (-0.49/0.58) | 0.88 | -0.07 (-0.55/0.42) | | 0.79 |

**Table S.9** P-values for slope differences in health behaviour trajectories following cessation of unpaid caregiving: comparisons with continuing caregivers and non-caregivers. Represents p-values for slope differences in health behaviour trajectories estimated from piecewise growth curve models, comparing individuals who exited unpaid caregiving with two propensity score-matched control groups: (1) those who continued caregiving, and (2) non-caregivers. Slope comparisons are made for two distinct time segments: the transition period (from one or two years before to the caregiving cessation year, “-1 to 0” or “–2 to 0”) and the post-transition period (from caregiving onset to four years after for physical activity, diet, and alcohol; and up to seven years for smoking). Outcomes include physical activity, fruit and vegetable consumption (diet), problematic drinking, and smoking. Models are stratified by caregiving intensity, place of care, sex, and age group. All estimates are derived from models fitted to matched samples and account for baseline differences through propensity score matching.

|  |  |  | Exit caregiving vs. non-caregiving | | |
| --- | --- | --- | --- | --- | --- |
| **Physical inactivity** | **(n exit)** |  | **Transition period**  **(“-2 to 0”)** |  | **Post-transition period**  **(“2 to 4”)** |
| Caregiving status | 3,340 | 0.09 (0.00/0.18) | 0.06 | -0.07 (-0.21/0.07) | 0.32 |
| Caregiving intensity | 3,307 |  |  |  |  |
| Low |  | 0.05 (-0.05/0.15) |  | 0.00 (-0.15/0.15) |  |
| High |  | 0.30 (0.10/0.49) | 0.009 | -0.39 (-0.68/-0.11) | 0.02 |
| Place of caregiving | 3,330 |  |  |  |  |
| Outside |  | 0.06 (-0.04/0.17) |  | 0.01 (-0.15/0.16) |  |
| Inside |  | 0.20 (0.03/0.37) |  | -0.28 (-0.53/-0.03) |  |
| Dual |  | 0.10 (-0.24/0.45) | 0.11 | -0.10 (-0.64/0.43) | 0.15 |
| **Transition*sex** | **3,340** |  | **0.23** |  | **0.17** |
| Male only | 1,247 | 0.12 (-0.03/0.27) | 0.10 | -0.12 (-0.33/0.10) | 0.30 |
| Female only | 2,093 | 0.07 (-0.05/0.19) | 0.26 | 0.00 (-0.18/0.18) | 0.99 |
| **Transition*age group** | **3,340** |  | **0.85** |  | **0.85** |
| Early adulthood (16-29) | 287 | -0.14 (-0.51/0.23) | 0.46 | 0.20 (-0.35/0.75) | 0.47 |
| Early mid-adulthood (30-49) | 874 | 0.09 (-0.09/0.27) | 0.33 | -0.02 (-0.30/0.26) | 0.89 |
| Late mid-adulthood (50-64) | 1,191 | 0.01 (-0.14/0.17) | 0.87 | 0.07 (-0.15/0.29) | 0.53 |
| Late adulthood (65+) | 988 | 0.11 (-0.06/0.28) | 0.21 | -0.19 (-0.44/0.06) | 0.14 |
| **Diet** | **(n exit)** |  | **Transition period**  **(“-2 to 0”)** |  | **Post-transition period**  **(“2 to 4”)** |
| Caregiving status | 3,363 | -0.01 (-0.10/0.09) | 0.81 | -0.02 (-0.14/0.10) | 0.77 |
| Caregiving intensity | 3,331 |  |  |  |  |
| low |  | 0.00 (-0.10/0.09) |  | -0.06 (-0.20/0.07) |  |
| high |  | -0.05 (-0.22/0.13) | 0.88 | 0.09 (-0.17/-0.01) | 0.46 |
| Place of caregiving | 3,353 |  |  |  |  |
| Outside |  | -0.01 (-0.11/0.08) |  | -0.08 (-0.21/0.06) |  |
| Inside |  | -0.02 (-0.16/0.12) |  | 0.14 (-0.07/0.35) |  |
| Dual |  | 0.03 (-0.30/0.36) | 0.98 | -0.22 (-0.75/0.32) | 0.23 |
| **Transition*sex** | **3,363** |  | **0.23** |  | **0.17** |
| Male only | 1,256 | 0.04 (-0.09/0.17) | 0.57 | -0.07 (-0.27/0.12) | 0.45 |
| Female only | 2,107 | -0.10 (-0.21/0.01) | 0.06 | 0.12 (-0.03/0.28) | 0.12 |
| **Transition*age group** | **3,363** |  | **0.82** |  | **0.90** |
| Early adulthood (16-29) | 287 | 0.15 (-0.16/0.46) | 0.34 | -0.33 (-0.76/0.10) | 0.14 |
| Early mid-adulthood (30-49) | 879 | -0.02 (-0.19/0.15) | 0.85 | 0.02 (-0.22/0.27) | 0.85 |
| Late mid-adulthood (50-64) | 1,197 | -0.03 (-0.17/0.11) | 0.68 | -0.05 (-0.26/0.15) | 0.61 |
| Late adulthood (65+) | 1,000 | 0.03 (-0.12/0.18) | 0.71 | -0.01 (-0.21/0.20) | 0.94 |
| **Problematic drinking** | **(n exit)** |  | **Transition period**  **(“-2 to 0”)** |  | **Post-transition period**  **(“2 to 4”)** |
| Caregiving status | 3,371 | -0.01 (-0.08/0.06) | 0.83 | -0.01 (-0.11/0.09) | 0.81 |
| Caregiving intensity | 3,337 |  |  |  |  |
| Low |  | -0.02 (-0.10/0.05) |  | -0.02 (-0.12/0.09) |  |
| high |  | -0.01 (-0.16/0.14) | 0.86 | -0.01 (-0.23/0.21) | 0.95 |
| Place of caregiving | 3,361 |  |  |  |  |
| Outside |  | 0.01 (-0.07/0.09) |  | -0.05 (-0.16/0.06) |  |
| Inside |  | -0.07 (-0.20/0.06) |  | 0.05 (-0.14/0.23) |  |
| Dual |  | -0.10 (-0.37/0.18) | 0.61 | 0.06 (-0.34/0.46) | 0.69 |
| **Transition*sex** | **3,371** |  | **0.23** |  | **0.78** |
| Male only | 1,259 | 0.00 (-0.11/0.12) | 0.96 | -0.02 (0.18/0.14) | 0.79 |
| Female only | 2,112 | 0.00 (-0.09/0.09) | 0.96 | -0.05 (-0.17/0.08) | 0.45 |
| **Transition*age group** | **3,371** |  | **0.23** |  | **0.11** |
| Early adulthood (16-29) | 288 | -0.12 (-0.40/0.16) | 0.41 | 0.10 (-0.30/0.50) | 0.63 |
| Early mid-adulthood (30-49) | 882 | 0.02 (-0.12/0.16) | 0.77 | -0.09 (-0.29/0.10) | 0.34 |
| Late mid-adulthood (50-64) | 1,199 | 0.13 (0.01/0.25) | 0.03 | -0.16 (-0.33/-0.01) | 0.05 |
| Late adulthood (65+) | 1,002 | -0.10 (-0.22/0.03) | 0.13 | 0.09 (-0.09/0.27) | 0.32 |
| **Smoking** | **(n exit)** |  | **Transition period**  **(“-1 to 0”)** |  | **Post-transition period**  **(“1 to 7”)** |
| Caregiving status | 5,385 | 0.07 (-0.01/0.14) | 0.08 | -0.08 (-0.16/-0.001) | 0.04 |
| Caregiving intensity | 5,338 |  |  |  |  |
| Low |  | 0.09 (0.01/0.17) |  | -0.10 (-0.19/-0.01) |  |
| high |  | 0.05 (-0.09/0.19) | 0.10 | -0.05 (-0.20/0.10) | 0.07 |
| Place of caregiving | 5,370 |  |  |  |  |
| Outside |  | 0.08 (-0.01/0.17) |  | -0.10 (-0.19/-0.01) |  |
| Inside |  | 0.03 (-0.09/0.16) |  | -0.03 (-0.16/0.10) |  |
| Dual |  | 0.15 (-0.154/0.43) | 0.25 | -0.21 (-0.52/0.10) | 0.13 |
| **Transition*sex** | **5,385** |  | **0.88** |  | **0.97** |
| Male only | 2,146 | 0.06 (-0.05/0.17) | 0.29 | -0.07 (-0.20/0.05) | 0.24 |
| Female only | 3,239 | -0.01 (-0.11/0.09) | 0.91 | -0.01 (-0.12/0.09) | 0.84 |
| **Transition*age group** | **5,385** |  | **0.27** |  | **0.09** |
| Early adulthood (16-29) | 866 | -0.01 (-0.19/0.16) | 0.88 | 0.03 (-0.17/0.22) | 0.79 |
| Early mid-adulthood (30-49) | 1,570 | 0.17 (0.04/0.30) | 0.01 | -0.20 (-0.33/-0.06) | 0.004 |
| Late mid-adulthood (50-64) | 1,776 | -0.07 (-0.20/0.07) | 0.33 | 0.06 (-0.08/0.19) | 0.41 |
| Late adulthood (65+) | 1,173 | 0.04 (-0.15/0.24) | 0.66 | -0.8 (-0.28/0.13) | 0.47 |

# S10: Modelling the Number of Cigarettes

## Distribution of number of cigarettes

**Figure S.14** Distribution of numbers of cigarettes smoked per day

; (Top Left) Histogram showing the distribution of the number of cigarettes smoked per day, including all participants; (Top Middle) Histogram showing the number of cigarettes smoked per day, excluding participants who reported zero cigarettes smoked; (Top Right) Histogram of the number of cigarettes smoked per day, excluding zeros and trimming values above the 80th percentile (capped at 80 cigarettes); (Bottom Left) Box plot of the number of cigarettes smoked per day, including participants with zero cigarettes smoked; (Bottom Right) Box plot of the number of cigarettes smoked per day, excluding participants who reported zero cigarettes smoked

## Modelling of the number of cigarettes

The number of cigarettes smoked per day was examined as a semi-continuous outcome to complement the binary smoking measure used in the main analysis. The distribution of this variable was highly right-skewed, with 87.1% of observations recorded as zero (non-smokers) and values ranging from 0 to 400 cigarettes per day. To address extreme values, the variable was trimmed at 80 cigarettes per day following graphical inspection (Figure S.14).

Both two-part models and zero-inflated count models have been recommended in the literature for modelling cigarette consumption data with excess zeros (Alfò and Maruotti, 2010; Pittman et al., 2020; Sheu et al., 2004). Two-part models separate the process into (1) the probability of smoking versus not smoking (logistic regression) and (2) the number of cigarettes consumed among smokers (linear regression), from which combined predictive marginal effects can be estimated. Zero-inflated negative binomial (ZINB) models, by contrast, jointly model the excess zeros and the overdispersed count distribution within a single framework.

To inform the model choice, several count-based approaches were compared, including standard Poisson, zero-inflated Poisson (ZIP), negative binomial, and zero-inflated negative binominal Poisson (ZINB) regression (Figure S.15). The ZINB model showed the best fit among the count-based approaches. However, when predicted trajectories from the ZINB model were compared with those from the two-part model within the piecewise growth curve framework, both approaches produced identical predicted marginal effects (Figure S.16). Given this equivalence in predicted outcomes, two-part models were selected for the analysis because they provide a more intuitive separation between smoking participation and smoking intensity, which aligns with the substantive interest in distinguishing whether caregiving transitions affect smoking initiation or consumption among existing smokers.

Two-part model vs. poisson regression techniques for numbers of cigarettes smoked

**Figure S.15** Comparison of Poison models for number of cigarettes

, including standard Poisson regression, zero-inflated Poisson (ZIP), negative binomial regression, and zero-inflated negative binomial (ZINB) regression

**Figure S.16** Poisson Comparison

of zero-inflated negative binomial Poisson model with two-part model, showing trajectories for the number of cigarettes for individuals who transition into caregiving (n=8,659) compared to those who remain non-caregivers (n=17,323).

## Caregiving Entry

In the full propensity score matched sample (n=25,976; 8,659 caregivers, 17,317 controls), transitioning into caregiving was associated with a significant increase in the predicted number of cigarettes smoked per day compared to matched non-caregivers (Figure S.17, top left panel). However, over 87% of observations were zero, meaning this result largely mirrors the binary smoking analysis reported in the main text.

To examine whether caregiving entry was also associated with changes in cigarette consumption among existing smokers, a subgroup analysis was restricted to participants who were smokers at baseline (n=4,278; 1,492 caregivers, 2,786 controls). No significant difference in cigarette consumption trajectories was found between those who transitioned into caregiving and those who did not (Figure S.17, top right panel). This suggests that caregiving entry is associated with the probability of smoking but not with the number of cigarettes smoked among existing smokers. When further stratified by caregiving intensity among baseline smokers (n=4,216; 1,141 low-intensity, 292 high-intensity caregivers, 2,783 controls), those transitioning into high-intensity caregiving appeared to increase their cigarette consumption, but this did not reach statistical significance (Figure S.17, bottom panel), likely due to the small number of high-intensity caregivers who smoked at baseline.

Overall, this analysis is consistent with the main findings on binary smoking status. Caregiving entry appears to be associated with an increased probability of smoking, while cigarette consumption among existing smokers remains largely unchanged unless individuals transition into high-intensity caregiving. Notably, the increase in cigarette consumption among high-intensity caregivers appeared to begin one year prior to the transition into caregiving.

**Figure S.17** Predicted number of cigarettes smoked per day before and after caregiving onset, based on two-part models and a propensity score matched sample**.** Top left panel: full sample (n=25,976; 8,659 caregivers, 17,317 controls). Top right panel: participants who were smokers at baseline (n=4,278; 1,492 caregivers, 2,786 controls). Bottom panel: participants who were smokers at baseline, stratified by caregiving intensity (n=4,216; 1,141 low-intensity [<20 hours/week] caregivers, 292 high-intensity [≥20 hours/week] caregivers, 2,783 controls). Time is centred on caregiving onset (time=0), with dashed lines marking the transition period. All participants were non-caregivers at baseline. Predictive average marginal effects (AME) are shown with 95% confidence intervals.

## Caregiving Exit

Among participants who were smokers at baseline and exited caregiving (n=996), the number of cigarettes smoked per day was modelled using two-part models and compared to two matched control groups: those who continued caregiving (n=306) and non-caregivers (n=2,074) (Figure 6.29). There was no evidence that caregiving exit was associated with a change in cigarette consumption. Compared to non-caregivers, the interaction term for the transition period was not statistically significant (p=0.77), nor was the post-transition period (p=0.69). Similarly, compared to continuing caregivers, no significant association was found during the transition period (p=0.15) or in the post-transition period (p=0.11), although exiters showed a suggestive decline in cigarette consumption relative to continuing caregivers that did not reach statistical significance.

These findings indicate that exiting caregiving was not associated with changes in the number of cigarettes smoked among individuals who were already smokers. Combined with the caregiving entry results, the sensitivity analysis supports the conclusion that the association between caregiving transitions and smoking operates primarily through smoking initiation and cessation rather than through changes in consumption intensity among existing smokers.

**Figure S.18** Number of cigarettes and exit - smoker at baseline

; average number of cigarettes smoked before and after caregiving exit across UKHLS waves 5 to 13 among participants who were smokers at baseline, comparing those who exited caregiving (n=996) with those who continued caregiving (n=306) and with non-caregivers (n=2,074). Time is centred around caregiving exit, with dashed lines marking transition points. All participants were smokers at baseline.

# S11: Sensitivity analyses using continuous and standardised outcomes

Physical inactivity and problematic drinking were defined using validated screening tools and established cut-offs, applied to the IPAQ physical activity measure and the AUDIT-C score. Even so, dichotomising an underlying continuous measure can obscure within-person variation and reduce statistical power. We therefore re-estimated the models for these two outcomes using their continuous forms, namely total moderate and vigorous physical activity minutes per week and the AUDIT-C score, to test whether the main associations held. Both outcomes were standardised to a mean of zero and a standard deviation of one. Standardisation places outcomes measured on different scales onto a common metric, so coefficients are expressed in standard deviation units and are comparable across behaviours. Smoking is a binary behaviour and diet was already analysed as a continuous variable, so this check does not apply to them. The continuous outcomes, in particular the AUDIT-C score, remained non-normally distributed. Linear models are robust to this given the large sample size, but the standardised coefficients are best read as a directional check on the binary findings rather than as precise effect estimates. The binary specifications remain the primary models reported in the main text.

**Table S.10** Fixed-effects sensitivity analyses for caregiving entry and exit, using continuous standardised outcomes (physical activity in total minutes and the AUDIT-C score)

| **Outcome** | **Transition** | **β (95% CI)** | **p** | **Observations** | **Individuals** |
| --- | --- | --- | --- | --- | --- |
| Physical activity (total minutes) | Entry | 0.07 (0.05/0.09) | <0.001 | 128,334 | 47,367 |
| Physical activity (total minutes) | Exit | −0.02 (−0.04/0.01) | 0.142 | 128,406 | 47,371 |
| AUDIT-C (score) | Entry | 0.02 (0.01/0.03) | <0.001 | 128,870 | 46,925 |
| AUDIT-C (score) | Exit | 0.00 (−0.01/0.01) | 0.966 | 128,978 | 46,929 |

**Table S.11** Piecewise growth-curve sensitivity analyses for transition into caregiving, using continuous standardised outcomes. Estimates are slope differences (caregivers vs matched controls) in SD units, with 95% CIs.

|  | **(n entering)** | **Transition period** | **p** | **Post-transition period** | **p** |
| --- | --- | --- | --- | --- | --- |
| **Physical activity (total minutes)** | | | | | |
| Caregiving status | 4,436 | 0.06 (0.02/0.10) | 0.006 | −0.06 (−0.12/0.00) | 0.045 |
| **AUDIT-C (score)** | | | | | |
| Caregiving status | 4,468 | 0.02 (−0.01/0.04) | 0.197 | −0.04 (−0.07/0.00) | 0.049 |

**Table S.12** Piecewise growth-curve sensitivity analyses for cessation of caregiving, comparing exiters with continuing caregivers and with non-caregivers, using continuous standardised outcomes.

|  | **(n exit)** | **Transition period** | **p** | **Post-transition period** | **p** |
| --- | --- | --- | --- | --- | --- |
| **Physical activity (total minutes)** | | | | | |
| Exit vs continuing caregivers | 3,340 | −0.07 (−0.15/0.00) | 0.053 | 0.11 (0.00/0.22) | 0.051 |
| Exit vs non-caregivers | 3,340 | −0.07 (−0.12/−0.02) | 0.009 | 0.08 (0.01/0.15) | 0.033 |
| **AUDIT-C (score)** | | | | | |
| Exit vs continuing caregivers | 3,371 | 0.00 (−0.04/0.05) | 0.867 | −0.01 (−0.07/0.05) | 0.760 |
| Exit vs non-caregivers | 3,371 | 0.01 (−0.02/0.04) | 0.493 | −0.02 (−0.06/0.02) | 0.355 |

**Figure S.19** Standardised piecewise growth curve trajectories for physical activity minutes and AUDIT-C score on the propensity score matched sample. As a sensitivity analysis to the dichotomised specifications, physical activity minutes and AUDIT-C scores were standardised (z-scores) and re-estimated. Top panels are centred on caregiving entry and compare non-caregivers with those entering caregiving. Bottom panels are centred on caregiving exit and compare those who exited caregiving with continued caregivers and with non-caregivers. Markers are model-predicted mean standardised scores; vertical bars are 95% confidence intervals. Time is centred on the caregiving transition (year 0).

# S12: Multiple imputation sensitivity analysis

As a sensitivity analysis, the piecewise growth curve models on the propensity score matched sample were re-estimated under multiple imputation to assess the influence of missing covariate data. Missing values were imputed using multiple imputation by chained equations (MICE). The number of imputations was determined using the formula and Stata routine proposed by Von Hippel (2020) which indicated that ten imputed datasets were sufficient across all outcomes. Only baseline variables used for the matching were entered into the imputation model including the baseline covariates, the caregiving transition variable, and the baseline measure of each outcome; the repeated, wave-specific outcome measures were not imputed. .(Leyrat et al., 2019) recommend including the outcome in the imputation model under matched designs, and we therefore included each outcome at baseline (pre-treatment) in the imputation model. Matching was then performed within each imputed dataset, the piecewise growth curve models were estimated in each, and the resulting estimates were combined across imputations using Rubin's rules (Rubin and Schenker, 1991).

The table below reports the pooled interaction terms between caregiving transition status and the piecewise slope components, which test whether trajectories differed between caregivers and their matched comparison group during the transition period and across the post-transition period. Results were broadly consistent with the complete-case piecewise models reported in the main analysis.

**Table S.13** Pooled slope differences (caregiving status × slope interactions) in health behaviour trajectories from piecewise growth curve models estimated on multiply imputed propensity score matched samples. Coefficients (β) are pooled interaction terms between caregiving transition status and the piecewise slope components, estimated on multiply imputed, propensity score matched samples and combined using Rubin’s rules. The pre-transition slope was held common across groups by design, as caregivers and comparisons were matched at baseline. The transition period runs from the wave before the transition to the transition year (two years for physical inactivity, fruit and vegetable consumption, and problematic drinking; one year for smoking). The post-transition period runs from the transition year to four years after (physical inactivity, fruit and vegetable consumption, problematic drinking) or up to seven years after (smoking). Binary outcomes (physical inactivity, problematic drinking, smoking) were modelled on the probability scale (average marginal effects); fruit and vegetable consumption was modelled as a continuous outcome (portions per day). Propensity score matching was repeated within each imputed dataset, so the matched comparison sample varies across imputations; a single matched n is therefore not reported. CI, confidence interval.

| **Outcome** | **Transition period** | | **Post-transition period** | |
| --- | --- | --- | --- | --- |
|  | **β (95% CI)** | **p** | **β (95% CI)** | **p** |
| **Transition into caregiving (vs. matched non-caregivers)** | | | | |
| Physical inactivity | -0.11 (-0.19/-0.04) | 0.004 | 0.14 (0.02/0.25) | 0.02 |
| Fruit and vegetable consumption | -0.02 (-0.09/0.05) | 0.62 | 0.03 (-0.08/0.15) | 0.59 |
| Problematic drinking | -0.03 (-0.09/0.03) | 0.34 | 0.05 (-0.04/0.13) | 0.29 |
| Smoking | 0.17 (0.10/0.24) | <0.001 | -0.19 (-0.26/-0.12) | <0.001 |
| **Exit caregiving vs. continuing caregiving** | | | | |
| Physical inactivity | 0.15 (0.00/0.30) | 0.05 | -0.17 (-0.40/0.07) | 0.16 |
| Fruit and vegetable consumption | -0.04 (-0.18/0.09) | 0.54 | 0.14 (-0.05/0.33) | 0.15 |
| Problematic drinking | 0.11 (0.00/0.22) | 0.04 | -0.15 (-0.31/0.01) | 0.07 |
| Smoking | -0.01 (-0.18/0.17) | 0.94 | 0.01 (-0.17/0.19) | 0.93 |
| **C. Exit caregiving vs. non-caregiving** | | | | |
| Physical inactivity | 0.08 (-0.01/0.17) | 0.10 | -0.06 (-0.19/0.08) | 0.43 |
| Fruit and vegetable consumption | -0.04 (-0.13/0.04) | 0.33 | 0.06 (-0.06/0.19) | 0.32 |
| Problematic drinking | 0.01 (-0.05/0.08) | 0.70 | -0.05 (-0.14/0.05) | 0.34 |
| Smoking | 0.02 (-0.06/0.10) | 0.63 | -0.03 (-0.11/0.05) | 0.52 |

**Figure S.20** Multiple imputation results: predicted health behaviour trajectories around caregiving onset, comparing non-caregivers with those entering caregiving. Time is centred on caregiving onset (year 0). Marginplots of the piecewise growth curve models estimated on the propensity score matched sample under multiple imputation by chained equations (m = 10 imputed datasets), with matching and estimation performed within each dataset and results pooled using Rubin's rules. For physical inactivity, problematic drinking and smoking, markers are average marginal predicted probabilities; for diet, markers are predicted mean daily portions of fruit and vegetables. Vertical bars are 95% confidence intervals. The "(m=10)" in panel titles denotes the number of imputations.

Figure S. 21 Multiple imputation results: predicted health behaviour trajectories around caregiving exit, comparing those who exited caregiving with those who continued caregiving. Time is centred on caregiving exit (year 0). MArginplots piecewise growth curve models estimated on the propensity score matched sample under multiple imputation by chained equations (m = 10 imputed datasets), with matching and estimation performed within each dataset and results pooled using Rubin's rules. For physical inactivity, problematic drinking and smoking, markers are average marginal predicted probabilities; for diet, markers are predicted mean daily portions of fruit and vegetables. Vertical bars are 95% confidence intervals. The "(m=10)" in panel titles denotes the number of imputations.

Figure S.22 Multiple imputation results: predicted health behaviour trajectories around caregiving exit, comparing those who exited caregiving with non-caregivers. Time is centred on caregiving exit (year 0). Marginplots of piecewise growth curve models estimated on the propensity score matched sample under multiple imputation by chained equations (m = 10 imputed datasets), with matching and estimation performed within each dataset and results pooled using Rubin's rules. For physical inactivity, problematic drinking and smoking, markers are average marginal predicted probabilities; for diet, markers are predicted mean daily portions of fruit and vegetables. Vertical bars are 95% confidence intervals. The "(m=10)" in panel titles denotes the number of imputations.

# Reference List for Supplement

Alfò, M., Maruotti, A., 2010. Two‐part regression models for longitudinal zero‐inflated count data. Canadian Journal of Statistics 38, 197–216.

Bruhn, J.G., Rebach, H.M., 2014. The Sociology of Caregiving / by John G. Bruhn, Howard M. Rebach., Clinical Sociology: Research and Practice. Springer Netherlands, Dordrecht. https://doi.org/10.1007/978-94-017-8857-1

Cefalu, M., Vegetabile, B.G., Dworsky, M., Eibner, C., Girosi, F., 2020. Reducing bias in difference-in-differences models using entropy balancing. arXiv.org.

Chou, C.-P., Yang, D., Pentz, M.A., Hser, Y.-I., 2004. Piecewise growth curve modeling approach for longitudinal prevention study. Computational Statistics & Data Analysis 46, 213–225.

Hainmueller, J., 2012. Entropy Balancing for Causal Effects: A Multivariate Reweighting Method to Produce Balanced Samples in Observational Studies. Political analysis 20, 25–46.

Imai, K., Ratkovic, M., 2014. Covariate balancing propensity score. Journal of the Royal Statistical Society Series B: Statistical Methodology 76, 243–263.

Leyrat, C., Seaman, S., White, I., Douglas, I., Smeeth, L., Kim, J., Resche-Rigon, M., Carpenter, J., Williamson, E., 2019. Propensity score analysis with partially observed covariates: How should multiple imputation be used?

McMullin, J., Schonberger, B., 2022. When good balance goes bad: A discussion of common pitfalls when using entropy balancing. Journal of Financial Reporting 7, 167–196.

Orsini, N., Greenland, S., 2011. A procedure to tabulate and plot results after flexible modeling of a quantitative covariate. The Stata journal 11, 1–29.

Pittman, B., Buta, E., Krishnan-Sarin, S., O’Malley, S.S., Liss, T., Gueorguieva, R., 2020. Models for analyzing zero-inflated and overdispersed count data: an application to cigarette and marijuana use. Nicotine and Tobacco Research 22, 1390–1398.

Rubin, D.B., Schenker, N., 1991. Multiple imputation in health‐are databases: An overview and some applications. Statistics in medicine 10, 585–598.

Sheu, M., Hu, T., Keeler, T.E., Ong, M., Sung, H., 2004. The effect of a major cigarette price change on smoking behavior in California: a zero‐inflated negative binomial model. Health Economics 13, 781–791.

Von Hippel, P.T., 2020. How many imputations do you need? A two-stage calculation using a quadratic rule. Sociological Methods & Research 49, 699–718.

Zhao, Q., Percival, D., 2017. Entropy Balancing is Doubly Robust. Journal of causal inference 5, 41–55.
